# Structural and Functional Characterization of the Bacterial Type III Secretion Export Apparatus

**DOI:** 10.1371/journal.ppat.1006071

**Published:** 2016-12-15

**Authors:** Tobias Dietsche, Mehari Tesfazgi Mebrhatu, Matthias J. Brunner, Patrizia Abrusci, Jun Yan, Mirita Franz-Wachtel, Charlotta Schärfe, Susann Zilkenat, Iwan Grin, Jorge E. Galán, Oliver Kohlbacher, Susan Lea, Boris Macek, Thomas C. Marlovits, Carol V. Robinson, Samuel Wagner

**Affiliations:** 1 University of Tübingen, Interfaculty Institute of Microbiology and Infection Medicine (IMIT), Section of Cellular and Molecular Microbiology, Tübingen, Germany; 2 Center for Structural Systems Biology (CSSB), University Medical Center Hamburg-Eppendorf (UKE) and German Electron Synchrotron Centre (DESY), Hamburg, Germany; 3 Institute of Molecular Biotechnology (IMBA), Vienna Biocenter (VBC), Vienna, Austria; 4 Research Institute of Molecular Pathology (IMP), Vienna Biocenter (VBC), Vienna, Austria; 5 Sir William Dunn School of Pathology, University of Oxford, Oxford, United Kingdom; 6 Department of Chemistry, University of Oxford, Oxford, United Kingdom; 7 University of Tübingen, Proteome Center Tübingen, Tübingen, Germany; 8 University of Tübingen, Center for BioinformaticsTübingen, Germany; 9 Yale University School of Medicine, Department of Microbial Pathogenesis, New Haven, Connecticut, United States of America; 10 Max Planck Institute for Developmental Biology, Biomolecular Interactions, Tübingen, Germany; 11 German Center for Infection Research (DZIF), Partner-site Tübingen, Tübingen, Germany; McMaster University, CANADA

## Abstract

Bacterial type III protein secretion systems inject effector proteins into eukaryotic host cells in order to promote survival and colonization of Gram-negative pathogens and symbionts. Secretion across the bacterial cell envelope and injection into host cells is facilitated by a so-called injectisome. Its small hydrophobic export apparatus components SpaP and SpaR were shown to nucleate assembly of the needle complex and to form the central “cup” substructure of a *Salmonella* Typhimurium secretion system. However, the *in vivo* placement of these components in the needle complex and their function during the secretion process remained poorly defined. Here we present evidence that a SpaP pentamer forms a 15 Å wide pore and provide a detailed map of SpaP interactions with the export apparatus components SpaQ, SpaR, and SpaS. We further refine the current view of export apparatus assembly, consolidate transmembrane topology models for SpaP and SpaR, and present intimate interactions of the periplasmic domains of SpaP and SpaR with the inner rod protein PrgJ, indicating how export apparatus and needle filament are connected to create a continuous conduit for substrate translocation.

## Author Summary

Many Gram-negative bacteria use type III secretion systems to inject bacterial proteins into eukaryotic host cells in order to promote their own survival and colonization. These systems are large molecular machines with the ability to transport proteins across three cell membranes in one step. It is believed that the only gated barrier of these systems lies in the bacterial cytoplasmic membrane but it was unclear so far how this gate looks like and of which components it is composed. Here we present evidence based on in depth biochemical and genetic characterization that an assembly of five SpaP proteins forms this gate in the cytoplasmic membrane of the type III secretion system of *Salmonella* pathogenicity island 1. We further show that one subunit each of the proteins SpaQ, SpaR, and SpaS are closely associated to the SpaP gate and may function in the gating mechanism, and that the protein PrgJ is attached to this gate on the outside to connect it to the hollow needle filament projecting towards the host cell. Our findings elucidate a hitherto ill-defined aspect of type III secretion systems and may help to develop novel antiinfective therapies targeting these virulence-associated molecular devices.

## Introduction

Type III secretion systems (T3SSs) are used by many Gram-negative bacterial pathogens and symbionts to translocate effector proteins in one step across the bacterial envelope and into eukaryotic host cells [1] where they modulate host cell physiology to promote bacterial survival and colonization [2]. The core of T3SSs is formed by the so-called injectisome, a macromolecular machine composed of up to 20 different proteins [1]. The base of the injectisome, consisting of an outer membrane secretin ring and two inner membrane ring components, anchors the system to the bacterial cell envelope [3]. A filamentous needle projects away from the base towards the host cell and serves as conduit for translocated effectors [4,5]. Five cytoplasmic proteins select and unfold the substrates, which are then handed over to the actual export apparatus [6,7] housed in a membrane patch at the center of the inner ring [8,9]. The five export apparatus components are thought to facilitate the actual secretion function of T3SSs, including energy coupling, membrane translocation, and substrate specificity switching [1]. Base, needle filament, and export apparatus are together also termed needle complex.

While analyses by X-ray crystallography and cryo electron microscopy have revealed the structure of most soluble components of injectisomes or of the related flagellar system [10,11], the structure and in particular the function of the hydrophobic transmembrane (TM) domains of the export apparatus components remain poorly defined. In the T3SS encoded within the pathogenicity island 1 (*S*PI-1) of *Salmonella enterica* serovar Typhimurium (*S*. Typhimurium), the export apparatus is composed of the proteins SpaP, SpaQ, SpaR, SpaS, and InvA in a 5:1:1:1:9 stoichiometry [12]. Of these components, InvA and SpaS are structurally and functionally best characterized: the atomic structures of their soluble cytoplasmic domains have been solved [13,14]. The large cytoplasmic domain of InvA (or its homologs) forms a nonameric ring with a central pore of about 50 Å in diameter [15] and has been proposed to play a role in substrate switching and translocation [16,17] while its 8 predicted TM helices have been proposed to serve in utilization of the proton motive force for secretion [18]. SpaS and its homologs play a role in switching of specificity from secretion of early to intermediate and late substrates [19]. Autocleavage of a highly conserved NPTH motif in the cytoplasmic domain of SpaS is required for this function, possibly to facilitate a high conformational flexibility of this domain for secretion of later substrates [20].

The substantially hydrophobic export apparatus components SpaP, SpaQ, and SpaR and their homologs were shown to be critical for assembly of the needle complex [9,21–23] and essential for secretion function [9,24] but their precise role in secretion is still unknown. It was suggested that SpaP and SpaR form the cup substructure of the needle complex [9]. Given the presumed central location of SpaP and SpaR at the center of the membrane patch of the needle complex and their substantial hydrophobicity, we hypothesized that these two proteins may constitute the actual substrate translocation pore of T3SSs in the bacterial inner membrane, a function that as yet has not been assigned to any T3SS component.

In this study, we have biochemically characterized a stable subcomplex formed by SpaP and SpaR, and mapped its place within the needle complex using *in vivo* photocrosslinking and complementary techniques. We show that an isolated complex of five SpaP and one SpaR forms a donut-shaped structure with an approximately 15Å wide recession at its center. Sole expression of the SpaP pentamer in the bacterial membrane allowed the permeation of compounds of 500 Da into the cytoplasm, suggesting that these proteins form a channel large enough for translocation of secondary structures. We further show that a complex of SpaP, SpaQ, SpaR, and SpaS assembles *in vivo* before incorporation into the needle complex base, and that these four export apparatus components form a compact assembly with multiple reciprocal interactions at TM helices three and four of the SpaP pentamer. We also present evidence that SpaP and SpaR interact on their periplasmic side with the inner rod protein PrgJ, which provides a basis to explain how the substrate translocation conduit is continuous from the export apparatus through the inner rod into the needle filament and suggests that the hitherto unaccounted electron density of the socket substructure is made of the periplasmic domains of SpaP and SpaR, together with PrgJ. In summary, we describe physical interactions among export apparatus components of bacterial T3SSs and identify the components that form its substrate translocation pore. This work will facilitate further structural and functional work on these machines and may help to develop novel antiinfective therapies targeting these virulence-associated molecular devices.

## Results

### SpaP and SpaR form a stable subcomplex of SpaP_5_R_1_ stoichiometry

**Fig 1:**
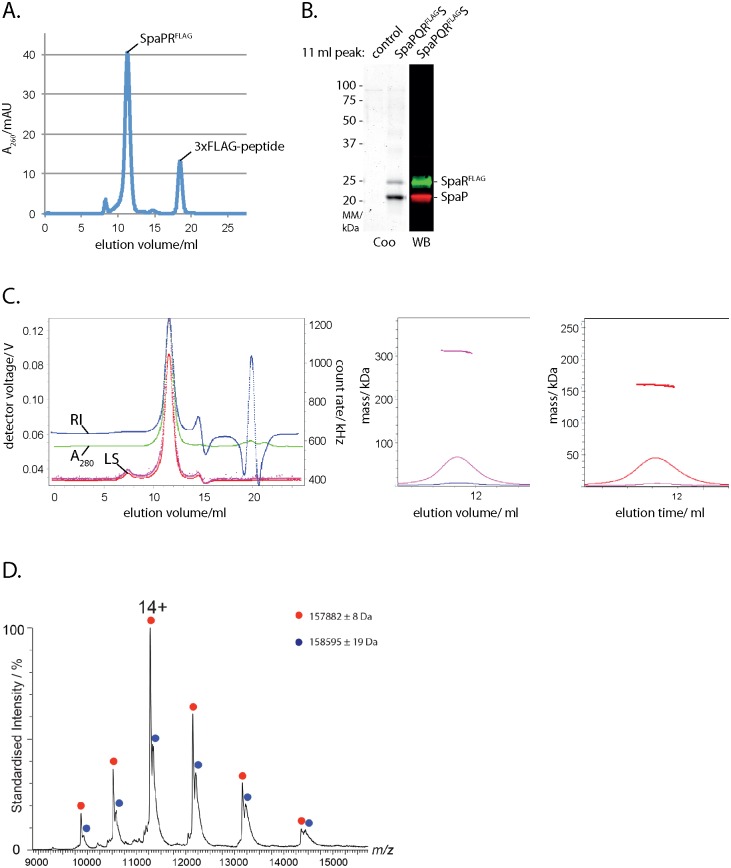
Isolation and stoichiometry analysis of the SpaPR subcomplex of the needle complex. (A) Elution profile of the purified SpaPR^FLAG^ complex run on a Superdex 200 10/300 GL column. The peaks corresponding to the SpaPR^FLAG^ complex and 3xFLAG peptide are indicated. (B) Coomassie-stained SDS PAGE gel of purified SpaPR^FLAG^ complex and of its FLAG-deficient control (left). Immunodetection of SpaP (green) and SpaR^FLAG^ (red) on Western blot from purified SpaPR^FLAG^ complex separated by SDS PAGE (right). (C) Traces of indicated detector signals from size exclusion chromatography—multi angle laser light scattering of purified SpaPR^FLAG^ complex (left). ASTRA-calculated mass profile of total components of peak of purified SpaPR^FLAG^ complex (polypeptides and detergent, middle). ASTRA-calculated mass profile polypeptide components of peak of purified SpaPR^FLAG^ complex (right). (D) Native mass spectrum of the SpaPR^STREP^ complex. Peak series corresponding to the SpaP:SpaR^STREP^ complex in a 5:1 ratio is marked in red, with the most abundant charge state (14+) indicated. The peak series marked in blue corresponds to the same SpaPR complex bound to a ligand with a mass of approximately 710 Da, indicative of an associated phospholipid. Note that the measured mass for SpaPR heterohexamer (157.882 kDa) is heavier than the theoretically calculated mass (157.280 kDa). Abbreviations: Coo: Coomassie stained, WB: Western blot, RI: refractive index, LS: light scattering.

We previously showed that a stable complex of SpaP and SpaR can be isolated from *S*. Typhimurium lacking the inner ring components PrgH and PrgK [9]. For further characterization, we expressed the *spaPQRS* operon in *Escherichia coli* and purified the SpaPR complex by immunoprecipitation of epitope-tagged SpaR. The isolated complex eluted as a sharp peak from a size exclusion chromatography column at an apparent size of 400 kDa (Fig 1A). Separation of the protein complex by SDS PAGE followed by Coomassie staining or Western blotting and immunodetection of SpaP and SpaR^FLAG^, respectively, showed that the complex contained more SpaP than SpaR (1B). Since the masses of membrane protein complexes deduced from analysis by size exclusion chromatography are skewed by the presence of bound detergent, we analyzed the fraction of protein and detergent contained in the isolated SpaPR complexes by size exclusion chromatography-multi angle laser light scattering. This analysis determined that the SpaPR peak was monodisperse, corresponding to a size of 311 kDa with a calculated protein content of 160 kDa (Fig 1C, S1 Table, S1 File), suggesting a total of 6 molecules of SpaP (25.2 kDa) and SpaR (31.7 kDa including C-terminal 3xFLAG tag). Given a mean error of 7% (S1 Fig), these data did not allow to distinguish whether the complex composition was 4 SpaP + 2 SpaR^FLAG^ (calc. 164 kDa) or 5 SpaP + 1 SpaR^FLAG^ (calc. 158 kDa). Native mass spectrometry was then performed to assess the exact stoichiometry of a purified SpaPR^STREP^ complex. A major species of complex produced peaks of 157.882 kDa and a minor species of 158.595 kDa. These masses are consistent with a stoichiometry of 5 SpaP and 1 SpaR^STREP^ (calculated molecular mass of 157.280 kDa) with bound phospholipids. In summary, these results show that the isolated SpaPR complex obtained from overexpression in the absence of other needle complex components has the same stoichiometry as SpaP and SpaR assembled into complete needle complexes [12] and indicates that the isolated SpaPR complex is a relevant functional module of the needle complex.

### Probing the placement of SpaP and SpaR in the needle complex by *in vivo* photocrosslinking

**Fig 2:**
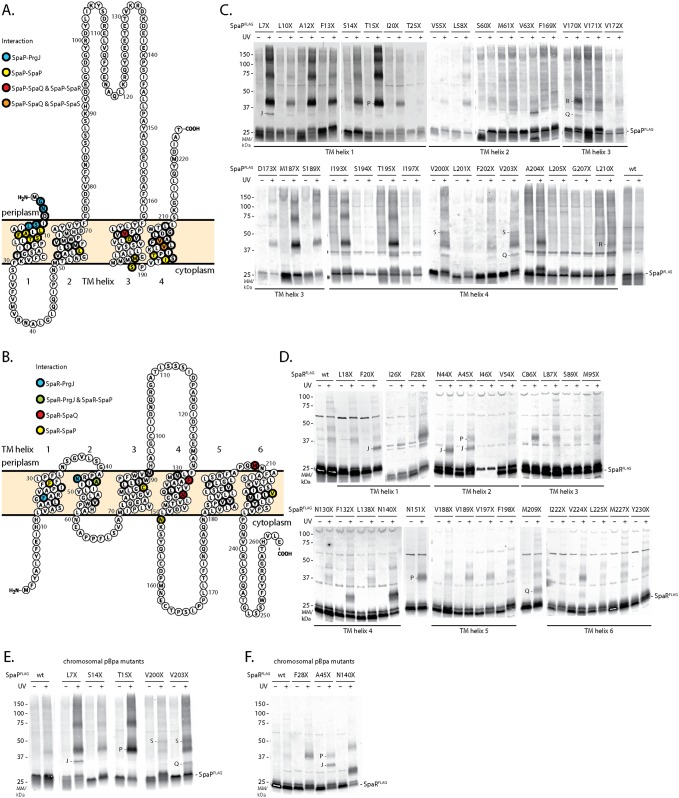
Screen of protein-protein interactions of SpaP and SpaR by *in vivo* photocrosslinking. (A) Protter visualization of SpaP presenting predicted TM topology, positions analyzed by *in vivo* photocrosslinking (thick stroke), and identity of interactions (colored). (B) As in (A) but showing SpaR. (C) Immunodetection of SpaP^FLAG^ on Western blots of crude membrane samples of *S*. Typhimurium expressing indicated plasmid-complemented SpaP-*p*Bpa mutants separated by SDS PAGE. *p*Bpa mutations are denoted as “X”. Each sample is shown with and without UV-irradiation to induce photocrosslinking of *p*Bpa to neighboring interaction partners. Since the running behavior of crosslinked proteins often deviates from the calculated mass due to incomplete unfolding and since membrane proteins like SpaP often show an aberrant running behavior, the position of a crosslink on a gel does not easily allow drawing direct conclusions on the size of the crosslinked adduct. Crosslinked proteins identified by mass spectrometry or Western blotting are indicated. Other highlighted interactions shown in A and B were based on comparable SDS PAGE band pattern. (D) As in (C) but showing SpaR complemented from a low-copy number plasmid expressing SpaPQR^FLAG^S. (E) As in (C) but expression of SpaP-*p*Bpa mutants from their chromosomal location. (F) As in (D) but expression of SpaR-*p*Bpa mutants from their chromosomal location. Abbreviations: J—PrgJ, P—SpaP, Q—SpaQ, S—SpaS.

To further validate the stoichiometry of SpaP and SpaR and to characterize the placing of this module within the assembled needle complex, we employed an *in vivo* photocrosslinking approach based on the genetically encoded UV-reactive amino acid *para*-benzophenylalanine (*p*Bpa) [25]. *p*Bpa was built into the predicted TM helices of SpaP and SpaR, respectively, so that possible interactions at every face of the predicted TM helices were sampled (Fig 2A and 2B). *spaP* or *spaPQRS* deletion mutants of *S*. Typhimurium were complemented with SpaP^FLAG^ or SpaPQR^FLAG^S containing *p*Bpa at selected positions and expressed from a low copy number plasmid. Complementation of T3SS function of these mutants was assessed by analyzing type III-dependent secretion of substrate proteins into the culture supernatant (S2 Fig). Crosslinking of *p*Bpa to nearby interactors was induced by UV irradiation of intact bacterial cells immediately after harvesting. Subsequently, crude membranes were isolated and crosslinking patterns were analyzed by SDS PAGE and immunodetection of the FLAG-tagged bait protein. Crosslinked adducts of different sizes were identified at various positions of SpaP and SpaR (Fig 2C and 2D). To exclude crosslinking artifacts resulting from plasmid-based complementation, *p*Bpa positions that produced representative crosslinking patterns were also introduced into the chromosome-encoded genes, and crosslinking was performed accordingly. Notably, for all tested chromosomal positions the quality of previously identified crosslinks could be confirmed while the efficiency of crosslinking improved in some cases, possibly due to a more efficient complex assembly achieved by expression of *p*Bpa-containing proteins from its native context (Fig 2E and 2F). To identify the nature of crosslinked adducts, needle complexes with *p*Bpa-containing SpaP^FLAG^ or SpaR^FLAG^ were purified, UV-irradiated, resolved by SDS PAGE, and gel slices of the positions of the crosslinks were analyzed by mass spectrometry (S3 Fig). This analysis identified crosslinks between SpaP and the export apparatus components SpaS and SpaQ, and between SpaP and the inner rod protein PrgJ. Furthermore, crosslinks between SpaR and SpaP, SpaQ, and PrgJ were also identified (S2 Table, Fig 2C and 2D). The detailed validation and interpretation of the crosslinking analysis is presented in the following three sections.

### Crosslinking of the SpaP pentamer

**Fig 3:**
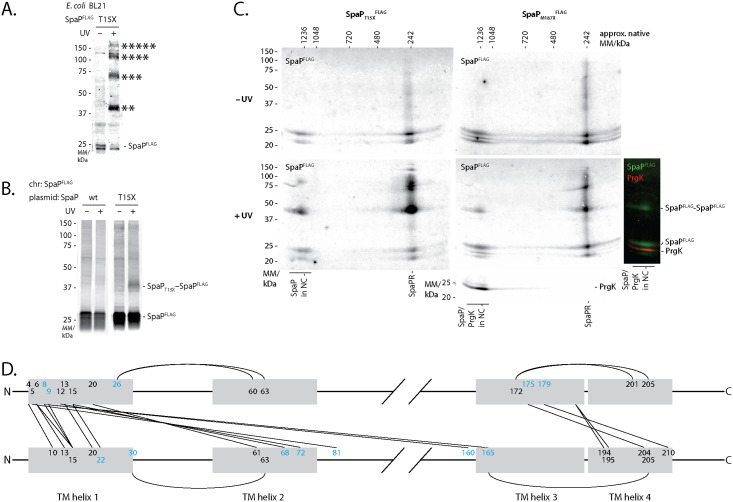
SpaP-SpaP interactions analyzed by *in vivo* photocrosslinking and sequence co-variation. (A) Immunodetection of SpaP^FLAG^ on Western blots of crude membrane samples of *E*. *coli* BL21 (DE3) expressing SpaP_T15X_^FLAG^ in the absence of all other T3SS components. The sample is shown with and without UV-irradiation to induce photocrosslinking of *p*Bpa to neighboring interaction partners. (B) Immunodetection of chromosome-encoded SpaP^FLAG^ on Western blots of crude membrane samples of *S*. Typhimurium expressing plasmid-encoded SpaP_T15X_. (C) Immunodetection of SpaP^FLAG^ and the inner MS ring protein PrgK on Western blots of crude membrane samples of *S*. Typhimurium expressing indicated SpaP-*p*Bpa mutants separated by 2-dimensional blue native/SDS PAGE. Full 2D gels are only shown for SpaP^FLAG^ scanned in the 800 nm channel. The 2D gel showing SpaP_M187X_^FLAG^ +UV has been re-probed with antibody for PrgK and scanned in the 700 nm channel. PrgK indicates the position of the assembled needle complex. An overlay of FLAG and PrgK signals is shown on the right. The relevant slice of the 700 nm image showing PrgK at 25 kDa and the overlay of both channels showing the needle complex-associated bands have been aligned to the corresponding 2D image. (D) Interaction map of SpaP. Lines indicate predicted interactions with a normalized coupling score > 0.8 (S3 Table) at positions with experimentally identified SpaP-SpaP crosslinks (at least from one side). Positions with experimentally observed SpaP-SpaP interactions are shown in black, target positions only predicted are shown in light blue. Grey shading indicates TM helices. Only positions within or in close proximity to TM helices are shown. Abbreviations: chr—chromosomal.

UV-irradiation of SpaP^FLAG^-containing *p*Bpa at positions L7, L10, A12, F13, S14, T15, M187, S189, I193, and T195 showed a ladder of crosslinks at 40 kDa, 70 kDa, 120 kDa, and 200 kDa (Fig 2C and 2E). We reasoned that this crosslink ladder might correspond to a homo-oligomeric crosslinking of the SpaP pentamer. Two further experimental results supported this hypothesis: First, crosslinking of SpaP_T15X_^FLAG^ expressed in *E*. *coli* in the absence of other T3SS components showed the same crosslink ladder (Fig 3A); and second, crosslinking plasmid-complemented SpaP_T15X_ in an *S*. Typhimurium strain with chromosome-encoded SpaP^FLAG^ also produced the 40 kDa FLAG-containing crosslink, which proved at least a bipartite SpaP_T15X_-SpaP^FLAG^ interaction (Fig 3B). Several of the SpaP *p*Bpa mutants that produced a ladder upon crosslinking (A12X, T15X, M187X, S189X, I193X) were non-functional (S2 Fig). Analysis of two of these *p*Bpa mutants (T15X and M187X) by 2-dimensional blue native/SDS PAGE indicated that the observed SpaP-SpaP interaction occurred between SpaP assembled into the complete needle complex as well as between SpaP molecules that had not yet been yet incorporated into this structure (Fig 3C). These results suggest that the loss of function of these mutants is unlikely due to improper folding or assembly but rather due to subtle conformational changes that alter their function.

Overall, these results indicate that TM helix one and to a smaller extent the cytoplasmic face of TM helix three and four are involved in protomer contacts in the SpaP homopentamer while only few homotypic interactions were observed at positions of TM helices two and three.

To cross-validate the experimental findings, we performed an independent prediction of SpaP-SpaP interactions based on analysis of sequence co-variation using the software EV couplings [26–28]. 27 of the experimentally tested SpaP positions were predicted to be involved in SpaP-SpaP interactions with a normalized coupling score >0.80 (S3 Table). 18 of the 27 experimentally tested positions yielded indications of SpaP-SpaP interactions, 2 positions were experimentally ambiguous because of very low expression levels of the mutated proteins, and 7 positions showed no signs of SpaP-SpaP interactions. As used, EV couplings does not distinguish between intra and intermolecular interactions. 6 of the predicted but experimentally negative positions are likely to be involved in intramolecular interactions, which are not detectable by the *in vivo* photocrosslinking approach used (Fig 3D). Many intermolecular interactions at experimentally tested SpaP positions were predicted to connect two TM helices 1 or TM helix 1 and 3 in a parallel fashion, and TM helices 1 and 2 or TM helices 3 and 4 in an antiparallel fashion (Fig 3D), supporting a SpaP topology as depicted in Fig 2A, while only the coupling prediction of SpaP_S189_ (to L11) opposed this model. Overall, the bioinformatic analysis supports our experimental results, strengthens the topology model of SpaP, and provides a first picture of the buildup of the SpaP pentamer.

### SpaQ, SpaR, and SpaS assemble independently of other T3SS components onto the SpaP pentamer and closely interact with each other

**Fig 4:**
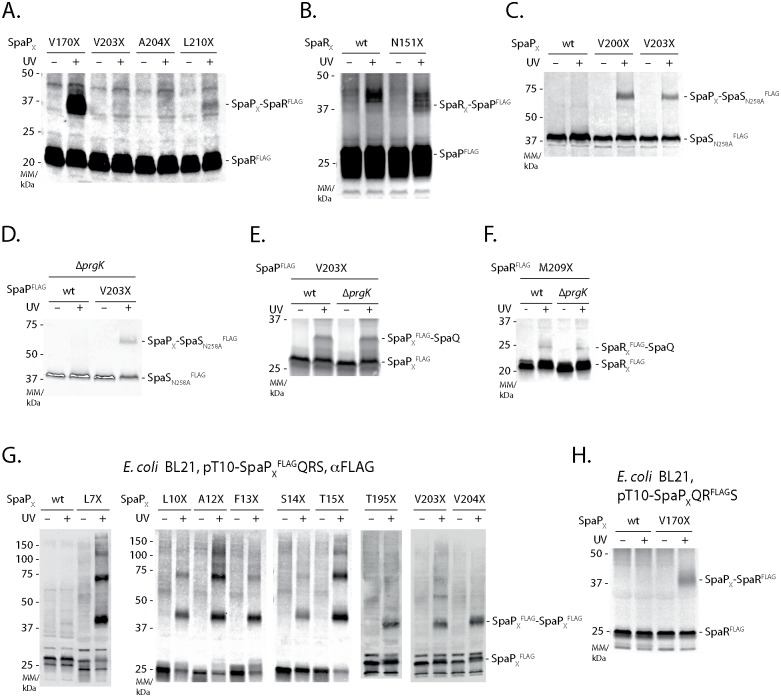
Interactions among the export apparatus components SpaP, SpaQ, SpaR, and SpaS. (A) Immunodetection of SpaR^FLAG^ on Western blots of SDS PAGE-separated crude membrane samples of Δ*spaPQRS S*. Typhimurium expressing indicated SpaP-*p*Bpa mutants from a pT10-*spaPQR*^FLAG^*S* plasmid. (B) Immunodetection of SpaP^FLAG^ on Western blots of SDS PAGE-separated crude membrane samples of Δ*spaPQRS S*. Typhimurium expressing indicated SpaR-*p*Bpa mutants from a pT10-*spaP*^FLAG^*QRS* plasmid. (C) Immunodetection of SpaS_N258A_^FLAG^ on Western blots of SDS PAGE-separated crude membrane samples of *S*. Typhimurium expressing indicated plasmid-complemented SpaP-*p*Bpa mutants. (D) As in (C) but assessing the SpaP-SpaS interaction in absence of the inner ring protein PrgK. (E) Immunodetection of SpaP^FLAG^ on Western blots of SDS PAGE-separated crude membrane samples of *S*. Typhimurium expressing chromosome-encoded indicated SpaP-*p*Bpa mutants in the presence or absence of the inner ring protein PrgK. (F) As in (E) but showing SpaR_M209X_^FLAG^. (G) Immunodetection of SpaP^FLAG^ on Western blots of crude membrane samples of *E*. *coli* BL21 (DE3) expressing indicated SpaP-*p*Bpa mutants together with SpaQRS to form the SpaPR complex. (H) As in (F) but expressing SpaP_V170X_QR^FLAG^S to reveal the SpaP-SpaR interaction in *E*. *coli*.

Mass spectrometry analysis of crosslinked SpaP and SpaR adducts produced evidence for multiple interactions among the export apparatus components SpaP, SpaQ, SpaR, and SpaS (Fig 2, S3 Fig, S2 Table). To validate these results by immunoblotting, we assayed the SpaP-SpaR as well as the SpaP-SpaS interactions by FLAG-tagging the target instead of the *p*Bpa-containing bait protein. We found that SpaP interacts with SpaR^FLAG^ through its residues V170 and L210 but not through V203 and A204 (Fig 4A) and that SpaR contacts SpaP^FLAG^ via its residue N151 (Fig 4B). Using an autocleavage-deficient FLAG-tagged variant of the switch protein SpaS, we could further validate interactions between SpaS and SpaP_V200X_/SpaP_V203X_ (Fig 4C). In summary, these crosslinking data indicate that, consistent with our previous report [12], 1 SpaQ, 1 SpaR, and 1 SpaS form a closely interconnected assembly that contacts SpaP at TM helix three (V170: SpaQ, SpaR) and TM helix four (V200/203: SpaQ, SpaS). The interaction of these four proteins seems to be integrated by SpaQ as this small protein makes contacts to all other three proteins (*in vivo* photocrosslinking-identified SpaS-SpaQ contacts communicated results of J. Monjarás Feria).

Previous results showed that SpaQ is critical for efficient formation of the needle complex base but due to technical limitations of the blue native PAGE approach used at the time, it was not clear whether assembly proceeds through a pre-assembled complex of all four minor export apparatus components before integration into the base or whether these components only interact upon base integration [9]. To examine the early events of the assembly of the T3SS export apparatus components, we probed the SpaP-SpaQ, SpaP-SpaS, and SpaR-SpaQ interactions identified by the crosslinking studies in strains deficient in the inner ring protein PrgK. These mutants are deffective for base assembly thus allowing to prove the requirement of a fully assembled base for the assembly of the export apparatus. Indeed, we detected SpaP-SpaQ and SpaP-SpaS interactions at SpaP_X203_ in the absence of PrgK (Fig 4D and 4E), and SpaR-SpaQ interactions at SpaR_X209_ (Fig 4F). SpaP-SpaP and SpaP_V170X_-SpaR crosslinks were also identified when plasmid-encoded SpaPQRS were expressed in *E*. *coli* BL21, lacking all other T3SS components (Fig 4G and 4H). Altogether, these results indicate that assembly of the export apparatus precedes and is independent of base assembly.

### The inner rod protein PrgJ locates close to the inner membrane and directly contacts the periplasmic domains of SpaP and SpaR

**Fig 5:**
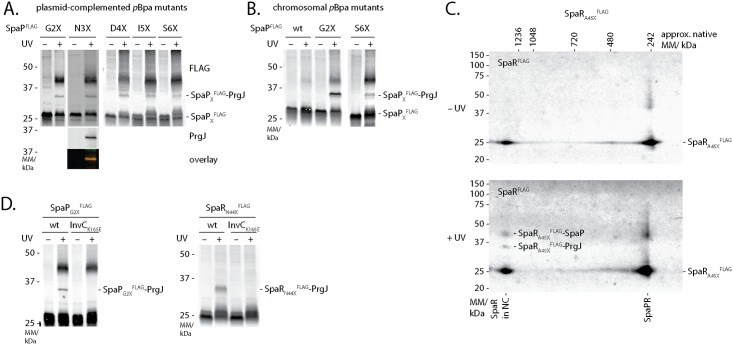
Interactions of SpaP and SpaR with the inner rod protein PrgJ. (A) Immunodetection of SpaP^FLAG^ on Western blots of crude membrane samples of *S*. Typhimurium expressing indicated plasmid-complemented SpaP-*p*Bpa mutants separated by SDS PAGE. The Western blot of SpaP_N3X_^FLAG^ was re-probed with PrgJ antibody to show the presence of SpaP and PrgJ in the same band. (B) Immunodetection as in (A) but detailing chromosome-encoded *p*Bpa-containing mutants of SpaP^FLAG^. (C) Immunodetection of SpaR^FLAG^ on Western blots of crude membrane samples of *S*. Typhiumurium expressing SpaR_A45X_^FLAG^ separated by 2-dimensional blue native/SDS PAGE. (D) Immunodetection of SpaP^FLAG^ or SpaR^FLAG^ on Western blots of SDS PAGE-separated crude membrane samples of *S*. Typhimurium expressing SpaP_G2X_^FLAG^ or SpaR_N44X_^FLAG^ in wild type or in InvC ATP-hydrolysis mutants, which are unable to secrete.

UV-irradiation of SpaP^FLAG^ with *p*Bpa at position L7 or SpaR^FLAG^ with *p*Bpa at positions F20, N44, and A45 resulted in an 8 kDa mobility shift of these proteins in SDS-PAGE (Fig 2C, 2D and 2E). Mass spectrometry analysis of the shifted bands identified PrgJ in both cases (S3 Fig, S2 Table). In an effort to characterize the extent of the SpaP-PrgJ interaction in more detail, we also noted the same mobility shift of SpaP after UV-irradiation of SpaP^FLAG^ with *p*Bpa at positions G2, N3, D4, I5, and S6, where crosslinked PrgJ was confirmed by immunodetection (Fig 5A). To rule out potential artifacts due to overexpression of the plasmid-borne constructs, we confirmed the crosslinks of SpaP_G2X_^FLAG^ and SpaP_S6X_^FLAG^ after expression from their native chromosomal context (Fig 5B). 2-dimensional blue native/SDS PAGE analysis of the crosslinks resulting from UV-irradiation of SpaR_A45X_^FLAG^ showed that the observed SpaR-PrgJ interaction is only observed when SpaR is incorporated into the needle complex (Fig 5C). Furthermore, SpaP-PrgJ as well as SpaR-PrgJ interactions were not observed in an ATPase activity-deficient InvC_K165E_ mutant, demonstrating that the detected interactions dependent on active type III secretion, which is consistent with the observation that incorporation of PrgJ into the needle complex and inner rod assembly require a functional type III secretion system (Fig 5D). Taken together, these results indicate that the periplasmic domains of SpaP and SpaR serve to anchor the inner rod protein PrgJ to the export apparatus, thus creating a continuous conduit for substrate translocation from the export apparatus to the needle filament.

### SpaP forms a donut-shaped structure with a pore conducive to molecules of 500 Da

The location of the SpaP_5_R_1_ complex at the center of the needle complex base, right underneath and connected to the filamentous conduit formed by the inner rod and needle proteins, suggests that this complex forms the T3SS’s substrate translocation pore in the bacterial inner membrane.

**Fig 6:**
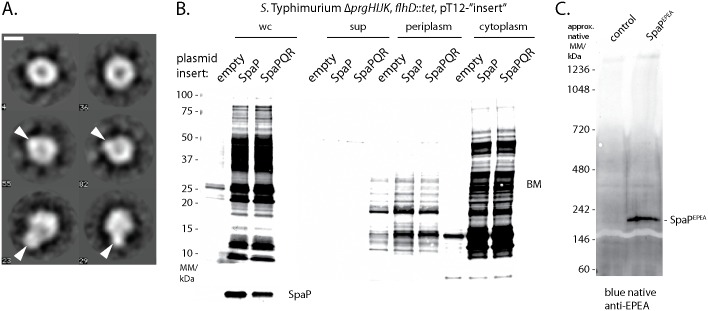
Visualization and characterization of the pore formed by SpaP and SpaR. (A) Six selected class averages (4, 23, 29, 36, 55, 82) of negative-stained isolated SpaPR complexes imaged by electron microscopy. The length of the scale bar represents 50 Å. The two class averages at the top represent the SpaP_5_ complex. Arrowheads in the class averages in the middle and at the bottom represent the anticipated position of SpaR on the SpaP_5_ ring. The complete picture of all class averages can be seen in S4 Fig. (B) Fluorescent streptavidin detection of SDS PAGE-separated biotin maleimide-labeled proteins of whole cell lysates, cell culture supernatant, periplasmic fraction, or cytoplasmic fraction of *S*. Typhimurium Δ*prgHIJK*, *flhD*::*tet* moderately overexpressing indicated proteins from a medium copy number plasmid (pT12). (C) Blue native PAGE and immunodetection of a high molecular weight complex formed by EPEA-tagged SpaP alone.

To obtain structural evidence for its putative pore-forming function, we analyzed the purified, negative-stained SpaPR^FLAG^ complex by electron microscopy. 11202 individual particles were classified and aligned into 91 class averages (S4 Fig). A number of class averages showed a symmetric, donut-shaped complex with an iconic recession at its center (Fig 6A). The diameter of these particles was about 80 Å and the diameter of the recession was about 15 Å. Other class averages showed a more asymmetric shape with an extra density outside of the ring-structure or a mushroom-like shape (Fig 6A). Even though the sample analyzed consisted of a homogeneous population of SpaPR^FLAG^ complexes, it cannot be ruled out that SpaP and SpaR^FLAG^ partly dissociated during sample preparation so that a mixture of SpaP_5_ and SpaP_5_R_1_ complexes was imaged, explaining the diversity of observed classes. It is therefore possible that the donut-shaped particles represent SpaP_5_ complexes and the asymmetric extension the SpaP-bound SpaR^FLAG^. Overall, the particles’ shape and dimensions conformed well with the structure of the cup region of assembled bases reported previously (3).

We reasoned that the recession at the center of the observed particles might represent the protein translocation pore of the T3SS. To probe the conducting properties of the SpaPR complex, we assessed its ability to allow the access of biotin maleimide (BM, molecular mass = 500 Da) into the bacterial cytoplasm, an approach that has been used previously to test the gating of the Sec-translocon [29]. The maleimide moiety of BM can only react with and biotinylate free thiol groups of cysteine residues of cytoplasmic proteins if BM can penetrate the inner bacterial membrane through a sufficiently large pore. The extent of biotinylation can then be detected on a Western blot by utilizing streptavidin. Strong BM labeling of proteins was observed in whole cell lysates when SpaPR or SpaP alone were overexpressed from a medium copy plasmid (Fig 6B). Cell fractionation of the expression host showed that only cytoplasmic proteins were differentially labeled by BM upon expression of SpaPR and SpaP, labeling of periplasmic proteins, however, was almost indistinguishable in control and expressing bacteria (Fig 6B). General lysis of the expression host could be ruled out to cause the observed phenotype as neither the cytoplasmic protein RNA polymerase nor the periplasmic maltose binding protein were observed in the culture supernatant of SpaPR or SpaP expressing bacteria (S5A and S5B Fig). Formation of a sizable, ungated pore by these complexes was also indicated by the strong impact even modest overexpression of SpaP and SpaPR had on the viability of the expression host (S5C Fig). Altogether, these results suggest that BM accessed the cytoplasm of the expression host through a pore formed by the expressed proteins. Since SpaP expression alone led to BM labeling of cytoplasmic proteins, it is conceivable that SpaP alone is sufficient to form the actual substrate translocation pore. In line with this idea, overexpressed SpaP^EPEA^ was observed to assemble into high molecular weight complexes when analyzed by blue native PAGE (Fig 6C), however, we were not able to isolate and investigate stable SpaP-only complexes. The access of 500 Da BM to the cytoplasm through the pore of the SpaP pentamer suggests a pore diameter of about 15 Å, which is consistent with the diameter of the recession observed by electron microscopy of the isolated SpaP_5_R_1_ complexes.

## Discussion

The export apparatus of bacterial T3SSs is its central unit that facilitates translocation of substrates across the bacterial inner membrane and likely the only gated barrier of these one-step secretion devices. While functions have been proposed for some export apparatus components, the components forming the actual substrate translocation pore in the bacterial inner membrane have not been defined.

In this study we present evidence that a homopentamer of the minor hydrophobic export apparatus component SpaP is a central component of the translocation pore in the inner membrane of the injectisome T3SS encoded by *Salmonella* pathogenicity island 1. We purified a stable complex of 5 SpaP and 1 SpaR that under electron microscopy exhibited a donut-like shape of about 80 Å in diameter and a 15 Å wide central recession. Expression of the components of this complex in *E*. *coli* rendered the bacterial cells permeable to 500 Da compounds, supporting the notion that it may work as translocation channel. Extensive mapping of protein-protein interactions of the TM domains of SpaP and SpaR by *in vivo* photocrosslinking revealed that SpaQ, SpaR, and SpaS form a compact assembly connected to the central pentamer formed by SpaP. We further demonstrated that assembly of this complex does not require its incorporation into the needle complex. We also detected crosslinks between SpaP and SpaR and the inner rod protein PrgJ showing that the inner rod makes direct contact with the export apparatus.

Previous analysis by blue native PAGE showed that SpaP and SpaR form stable complexes in an *S*. Typhimurium mutant unable to assemble the needle complex [9]. We now present evidence based on size-exclusion chromatography-multi angle laser light scattering and native mass spectrometry that this complex is composed of 5 SpaP and 1 SpaR. The stoichiometry of the isolated SpaP_5_R_1_ complex is consistent with the stoichiometry of SpaP and SpaR in the context of a fully assembled needle complex [12], which indicates that the isolated complex represents a relevant intermediate of needle complex assembly. This notion is further supported by the good match of the dimensions of the observed SpaPR complex with the dimensions of the cup substructure of the needle complex [30], which we previously showed to be composed of SpaP and SpaR [9]. Electron micrographs of the isolated SpaP_5_R_1_ complex and BM permeation experiments suggested a pore size of the substrate translocation channel of about 15 Å. Within the range of uncertainty, this diameter conforms with the 10 Å that were reported for the dimensions of the channel of an assembled *S*. Typhimurium *S*PI-1 needle complex containing a trapped translocation intermediate [5]. A tight seal during substrate translocation is expected to be important for T3SS to avoid leakage of ions through the open pore, so it is conceivable that the pore diameter closely resembles the dimensions of extended polypeptides or alpha helices. However, a larger pore diameter in its fully open state cannot be excluded given that the herein investigated isolated SpaP_5_R_1_ complex most certainly lacks the necessary elements for gating of the pore.

We detected extensive crosslinks of up to five consecutive SpaP at TM helix one and at the cytoplasmic face of TM helices three and four, suggesting that these regions form the major contact area between protomers of the SpaP pentamer. This notion was supported by results of a sequence co-variation-based prediction of residue-residue interactions of SpaP. The formation of these crosslinks was independent of the presence of other needle complex components, supporting the notion that the SpaP pentamer nucleates assembly of the needle complex. Interestingly, the presence of SpaP pentamer crosslinks at TM helices three and four correlated with secretion defects of the respective *p*Bpa mutants, a phenomenon also seen for SpaP_A12X_ and SpaP_T15X_. The secretion defect was not due to defects in their incorporation into assembled needle complexes, suggesting that these residues may play a critical role in protein translocation.

**Fig 7:**
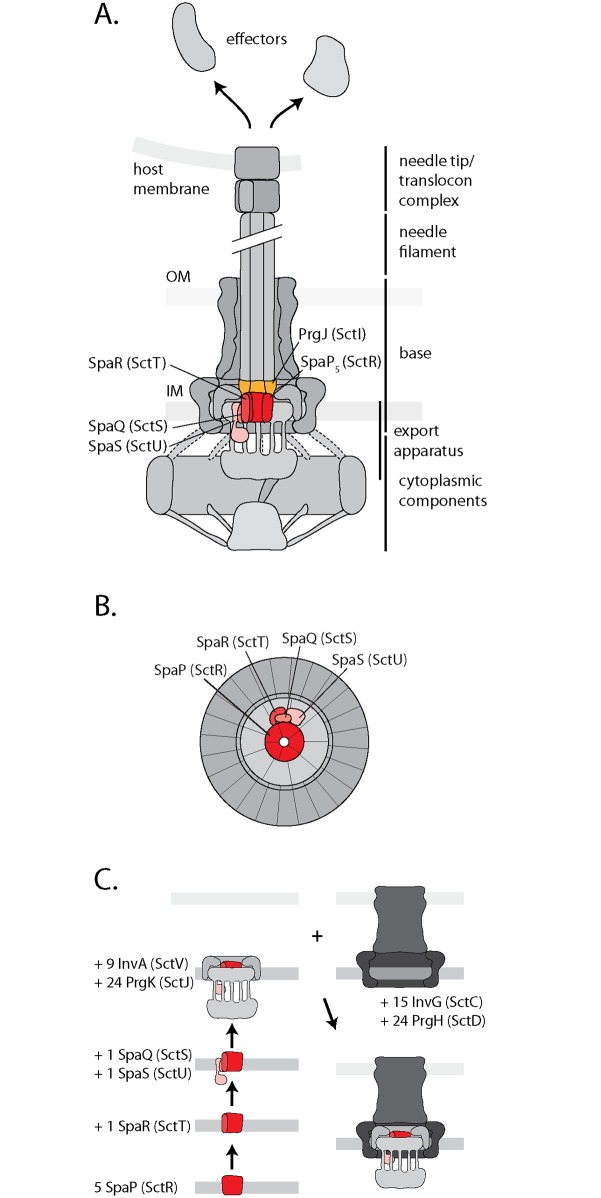
Models of SpaP, SpaR, SpaQ, SpaS, and PrgJ in the T3SS needle complex and its assembly. (A) Model of the central SpaP complex with surrounding export apparatus components SpaQ, SpaR, and SpaS, and direct connection to the inner rod formed by PrgJ. These results suggest that SpaP, SpaR, and PrgJ form the socket structure on the periplasmic side of the inner membrane patch of the base. (B) Model of a view of the membrane patch of the needle complex from the cytoplasmic side highlighting SpaP, SpaQ, SpaR, and SpaS. (C) Model of needle complex assembly. The unified Sct nomenclature [23] is shown in parenthesis.

The recently reported stoichiometry of SpaP, SpaQ, SpaR, and SpaS of 5:1:1:1 [12] suggests that these export apparatus components form an asymmetric assembly within the needle complex. We show here that SpaQ, SpaR, and SpaS contact the SpaP pentamer at its TM helices three and four. We further demonstrate that SpaQ interacts with SpaP and SpaR. These observations, together with the observation that a fusion of SpaR and SpaS homologs retains function [31], suggest that SpaQ, SpaR, and SpaS are not wrapped around the SpaP pentamer but form a compact assembly at one side of SpaP, with SpaQ as the central component that makes contacts to all other components (Fig 7A and 7B). Besides SpaR’s contribution in anchoring the inner rod protein PrgJ, the assembly formed by SpaQ, SpaR, and SpaS might also facilitate gating of the SpaP pore, a critical aspect to prevent detrimental effects of nutrient and ion leakage across the bacterial inner membrane.

The assessment of the dependence of crosslinks between SpaP, SpaQ, SpaR, and SpaS on the presence of the inner ring protein PrgK allowed us to refine a model for the early steps of export apparatus assembly (Fig 7C). We propose that assembly starts with the formation of the SpaP pentamer. This initially unstable complex is stabilized upon binding SpaR. The high stability of the resulting SpaP_5_R_1_ intermediate suggests that this complex is the major nucleus of further needle complex assembly. Next, SpaQ and SpaS associate with the SpaP_5_R_1_ complex but presumably with weaker affinity since this complex could only be captured after *in vivo* crosslinking. InvA would then be recruited to the SpaPRQS complex although it is not clear whether its recruitment occurs prior or after this complex initiates the assembly of the needle complex rings. Subsequently, association of the outer membrane secretin InvG and the inner ring protein PrgH would lead to formation of the completed base-export apparatus holo-complex [21,23].

Beyond interactions among the export apparatus components, we also identified crosslinks between the periplasmic domains of SpaP and SpaR and the inner rod protein PrgJ. The close interaction of SpaP, SpaR, and PrgJ is likely to create a continuous conduit for substrate translocation, where PrgJ might serve as an adapter to connect the flat translocation pore of the inner membrane with the helical needle filament. Analysis of the needle complex by cryo-electron microscopy revealed a central juxtamembrane structure at the periplasmic interior of the base, which was termed socket [30], however, no protein could be assigned to contribute to this density. Our results suggest that the socket is composed of the periplasmic parts of SpaP and SpaR, together with the inner rod protein PrgJ. The mass of six PrgJ [12] and the periplasmic domains of five SpaP and one SpaR could well account for the observed density of the socket structure. Our observation now opens the door for further investigations of the relevance of the export apparatus-PrgJ interaction for needle length control, substrate specificity switching, and host cells sensing, functional roles that were suggested for PrgJ [32,33].

The positions of SpaP and SpaR that interact with PrgJ also help to consolidate the TM topology models of these two export apparatus proteins. SpaP is predicted to contain four TM helices (Fig 2A) and the presence of a cleavable signal sequence in flagellar homologs suggests an N-out/C-out TM orientation [34]. This model is supported by the interaction between the N-terminus of SpaP and the periplasmic inner rod detected in this study. Further support for this topology model comes from the presented sequence co-variation-based analysis of SpaP residue-residue interactions, which strongly predicted antiparallel interactions between TM 1 and 2, and between TM 3 and 4 (Fig 3D). The TM topology predictions of SpaR and its homologs are very uncertain, ranging from five to eight TM helices with mostly N-out orientation (Fig 2B, S6 Fig) [34,35]. A C-in orientation, on the other hand, was suggested based on the report of a functional protein fusion of the flagellar SpaR and SpaS homologs of *Clostridium*, given that the N-terminus of SpaS and its homologs is strongly predicted to reside in the cytoplasm [31,35,36]. Here we presented interactions of SpaR F20, N44, and A45 with the periplasmic protein PrgJ. These residues are predicted to be located within SpaR’s first two TM helices, however, our results rather suggest a periplasmic localization of this part of SpaR. This notion is supported by rather high ΔG values for membrane partitioning of the predicted TM helices one, two, and four (S6B Fig), so that a SpaR model comprising an N-out/C-in topology with only three TM helices is conceivable (S6C Fig).

In summary, we have presented evidence that a pentamer of SpaP forms the substrate translocation pore of T3SSs in the bacterial inner membrane. We show that this pentamer closely interacts with the export apparatus components SpaQ, SpaR, and SpaS in the plane of the membrane, an accessory assembly that may facilitate gating of the export pore. We further show that SpaP and SpaR intimately contact the periplasmic inner rod protein PrgJ and propose that the inner rod serves as an adapter to connect the flat export pore and the helical needle filament, thus creating a continuous conduit for substrate translocation from the bacterial cytoplasm into the host cell.

## Materials and Methods

### Materials

Chemicals were from Sigma-Aldrich unless otherwise specified. Detergent n-dodecyl-maltoside (DDM) was from Affimetrix-Anatrace. para-benzophenylalanine was from Bachem. SERVA Blue G and SERVAGel TG PRiME 8–16% precast gels were from Serva. NativePAGE Novex Bis-Tris 3–12% gels were from Life Technologies. Primers are listed in S5 Table and were synthetized by Eurofins and Integrated DNA Technologies. Polyclonal rabbit anti-MBP antibody were from New England Biolabs. Monoclonal mouse anti-RNApol antibody was from BioLegend. Monoclonal M2 anti-FLAG antibody, M2 anti-FLAG agarose beads, and 3xFLAG peptide were from Sigma-Aldrich. CaptureSelect-biotin, Streptavidin DyLight 800, and secondary antibodies goat anti-mouse IgG DyLight 800 conjugate and goat anti-rabbit IgG DyLight 680 conjugate were from Thermo-Fisher.

### Bacterial strains and plasmids

Bacterial strains and plasmids used in this study are listed in S4 Table. Primers for construction of strains and plasmids ere listed in S5 Table. The position and sequence of epitope tags introduced into SpaP, SpaR, and SpaS is shown in S7 Fig. All *Salmonella* strains were derived from *S*. Typhimurium strain SL1344 [37]. Bacterial cultures were supplemented as required with streptomycin (50 μg/mL), tetracycline (12.5 μg/mL), ampicillin (100μg/mL), kanamycin (25 μg/mL), or chloramphenicol (10 μg/mL).

### Expression and purification of SpaPR complex

The SpaP, and SpaPR complexes were expressed in *E*. *coli* BL21 (DE3) from rhamnose-inducible medium copy number plasmids encoding SpaP^EPEA^, SpaPQR^FLAG^S, or SpaPQR^STREP^, respectively. Expression was autoinduced by over night growth at 37°C in TB medium. Bacterial cells were harvested, crude membranes purified as described previously [9], and membrane proteins were extracted with 1% DDM in PBS. After removal of unsolubilized material by ultracentrifugation for 30 min at 100.000 x g, complexes were immunoprecipitated according to the manufacturers instructions using CaptureSelect affinity gel for SpaP^EPEA^, M2 anti-FLAG agarose beads for SpaPR^FLAG^, and Strep-Tactin sepharose (IBA) for SpaPR^STREP^. Complexes were natively eluted with 150 ng/ml SEPEA or 3xFLAG peptides, respectively, or with 2.5 mM desthiobiotin, each in PBS/0.04% DDM. The SpaP^EPEA^ and the SpaPQR^FLAG^S, complexes were subsequently purified by anion exchange (Mono Q 5/50 GL, GE), while this step was omitted for the SpaPQR^STREP^ complex. Samples were further purified by size exclusion (Superdex 200 10/300 GL, GE) chromatography, and concentrated to 1 mg/ml using Amicon Ultra 100 k cutoff spin concentrators (Merck Millipore). Purified SpaP and SpaPR complexes were stored in liquid nitrogen until further use.

### Size exclusion chromatography—multi angle laser light scattering analysis

The detergent and polypeptide content of the purified SpaPR^FLAG^ complex in PBS/0.04% DDM was determined by size exclusion chromatography—multi angle laser light scattering and analysis by the ASTRA software (Wyatt, Santa Barbara, CA) as previously described [38].

### Native mass spectrometry of isolated native SpaPR complex

Purified SpaPR^STREP^ complex was concentrated to 20 μM in PBS/0.04% DDM, and buffer exchanged to 250 mM ammonium acetate, pH 7.5, complemented with 0.01% polyoxyethylene(9)dodecyl ether (C12E9) prior to native mass spectrometry analysis. Buffer exchange was carried out using Amicon Ultra 0.5 ml centrifugal filters with a 100-kDa cut-off (Millipore UK Ltd, Watford UK). Mass measurements were carried out on a Synapt G1 HDMS (Waters Corp., Manchester, UK) Q-ToF mass spectrometer [39]. The instrument was mass calibrated using a solution of 10 mg/ml cesium iodide in 250 mM ammonium acetate. 2.5 μL aliquots of samples were delivered to the mass spectrometer by means of nano-electrospray ionization via gold-coated capillaries, prepared in house [40]. Instrumental parameters were as follows: source pressure 6.0 mbar, capillary voltage 1.40 kV, cone voltage 150 V, trap energy 200 V, transfer energy 10 V, bias voltage 5 V, and trap pressure 1.63x10-2 mbar.

### Membrane protein topology prediction

SpaP and SpaR TM topology was predicted using TOPCONS (http://topcons.cbr.su.se) [41]. The extent of the hydrophobic regions constituting TM helices was predicted using dGpred full portein scan (http://dgpred.cbr.su.se) [42] setting the minimal helix length to 18 and the maximal helix length to 31 aa. For visualization, the online tool PROTTER (http://wlab.ethz.ch/protter/start/) was used [43].

### Secretion assay

Analysis of type III-dependend secretion of proteins into the culture medium was carried out as described previously [20].

### Immunoblotting

For protein detection, samples were subjected to SDS PAGE using SERVAGel TG PRiME 8–16% precast gels, transferred onto a PVDF membrane (Bio-Rad), and probed with primary antibodies anti-SipB, anti-InvJ, anti-PrgJ, anti-SpaP, anti-MBP, anti-RNApol, and M2 anti-FLAG. Secondary antibodies were goat anti-mouse IgG DyLight 800 conjugate and goat anti-rabbit IgG DyLight 680. EPEA-tagged SpaP was visualized using CaptureSelect-biotin anti C-Tag conjugate and Streptavidin DyLight 800. Scanning of the PVDF membrane and image analysis was performed with a Li-Cor Odyssey system and image Studio 2.1.10 (Li-Cor).

### *In vivo* photocrosslinking

*S*. Typhimurium strains were grown at 37°C in LB broth supplemented with 0.3 M NaCl with low aeration to enhance expression of genes of *S*PI-1. For *in vivo* photocrosslinking of SpaP^FLAG^ in *Escherichia coli* BL21 (DE3), bacteria were cultured at 37°C in LB broth. Cultures were supplemented with 500 μM rhamnose to induce expression of SpaP^FLAG^, SpaP^FLAG^QRS or SpaPQR^FLAG^S from low copy number pTACO10 plasmids [9]. To boost general *S*PI-1 expression, *S*. Typhimurium strains were transformed with pBAD24-hilA. Expression of the *S*PI-1 master regulator HilA was induced by addition of 0,05% arabinose to the cultures. Additionally the cultures were supplemented with the artificial amino acid para-benzoyl phenyl alanine (pBpa) to a final concentration of 1 mM and afterwards incubated for 5.5 h. 2 ODU of bacterial cells were harvested and washed once with 1 mL cold PBS. Cells were resuspended in 1 mL PBS and transferred into 6-well cell culture dishes. UV irradiation with λ = 365 nm was done on a UV transilluminator table (UVP) for 30 min.

### Crude membrane preparation

10 OD units of bacterial lysates of *S*. Typhimurium or *E*. *coli*, respectively, were resuspended in 750 μl buffer K (50 mM triethanolamine, pH 7.5, 250 mM sucrose, 1 mM EDTA, 1 mM MgCl_2_, 10 μg/ml DNAse, 2 mg/mL lysozyme, 1:100 protease inhibitor cocktail), and incubated for 30 min on ice. Samples were bead milled and beads, unbroken cells and debris were removed by centrifugation for 10 min at 10.000 x g and 4°C. Crude membranes contained in the supernatant were precipitated by centrifugation for 45 min at 55,000 rpm and 4°C in a Beckman TLA 55 rotor. Pellets containing crude membranes were frozen until use.

### Blue native PAGE

1-dimensional blue native PAGE and 2-dimensional blue native/SDS PAGE of crude membranes was carried out as previously described [9].

### Needle complex purification

*S*. Typhimurium Δ*spaP* or Δ*spaPQRS* mutants, respectively, transformed with pSUP, pSB3292, and pSB3398-based rhamnose-inducible low copy number plasmids containing SpaP^FLAG^ amber mutants or SpaPQRS with SpaR^FLAG^ amber mutants, respectively, were grown in 200 ml LB broth supplemented with 0.3M NaCl, 1 mM *p*Bpa, 500 μM rhamnose, 0.02% arabinose, and appropriate antibiotics for 5 h at low aeration to express *S*PI-1 and assemble needle complexes. Purification of needle complexes was carried out as published previously [4,20,12] but LDAO was replaced by DDM (0.7% for lysis/extraction, 0.1% for maintenance) for lysis of cells and extraction of needle complexes throughout the protocol. Furthermore, an initial concentration of 35% (wt/vol) of CsCl was used to prepare the gradient. Purified needle complexes containing SpaP^FLAG^ or SpaR^FLAG^ with *p*Bpa at desired positions were irradiated with UV light (365 nm) for 30 min to induce photocrosslinking to nearby proteins. Samples were subsequently analyzed by SDS PAGE, Western blotting, and immunodetection with M2 anti-FLAG antibodies. For MS analysis of crosslinked adducts, gel pieces at positions of observed crosslinks of *p*Bpa-containing and control samples were cut out of Coomassie-stained SDS PAGE gels and subjected to in gel digestion.

### Protein in-gel digestion for analysis of crosslinked interaction partners

For identification of crosslinked proteins, the area of a Coomassie-stained gel corresponding the position of the crosslinked band detected by Western blotting were excised and in-gel digested with trypsin [44]. For a better recovery, remaining proteins in the gel were again subjected to another tryptic digestion step. After each step extracted peptides were desalted using C_18_ StageTips [45]. Corresponding eluates were combined and subjected to LC-MS/MS analysis.

### Mass spectrometry for analysis of crosslinked interaction partners

LC-MS/MS analyses were performed on an EasyLC II nano-HPLC (Proxeon Biosystems) coupled to an LTQ Orbitrap Elite mass spectrometer (Thermo Scientific) as decribed elsewhere [46] with slight modifications: The peptide mixtures were injected onto the column in HPLC solvent A (0.5% acetic acid) at a flow rate of 500 nl/min and subsequently eluted with a 106 min gradient of 5–33% HPLC solvent B (80% ACN in 0.5% acetic acid). During peptide elution the flow rate was kept constant at 200 nl/min. For proteome analysis, the 20 (Orbitrap Elite) most intense precursor ions were sequentially fragmented in each scan cycle using collision-induced dissociation (CID). In all measurements, sequenced precursor masses were excluded from further selection for 90 s. The target values for MS/MS fragmentation were 5000 charges and 10^6^ charges for the MS scan.

### Mass spectrometry data processing for analysis of crosslinked interaction partners

The MS data were processed with MaxQuant software suite v.1.2.2.9 as described previously [47–49] with slight modifications. Database search was performed using the Andromeda search engine [48], which is part of MaxQuant. MS/MS spectra were searched against a target database consisting of 10,152 protein entries from *S*. Typhimurium and 248 commonly observed contaminants. In database search, full tryptic specificity was required and up to two missed cleavages were allowed. Carbamidomethylation of cysteine was set as fixed modification, protein N-terminal acetylation, and oxidation of methionine were set as variable modifications. Initial precursor mass tolerance was set to 6 parts per million (ppm) and at the fragment ion level 0.5 dalton (Da) was set for CID fragmentation. The MS data have been deposited to the ProteomeXchange Consortium (http://proteomecentral.proteomexchange.org) via the PRIDE partner repository with the data set identifier PXD005028.

### EVfold coupling analysis

Sequence co-variation analysis was performed using EVcouplings [26–28] with pseudo-maximum likelihood approximation [50–52]. The multiple sequence alignment used as input for the model inference was created by jackhmmer 3.1 [53] (5 iterations) using the full sequence of Salmonella SpaP (UniProt: SPAP_SALTY, residues 1–224) as query against the November 2015 release of the UniProt Reference Cluster database (UniRef100) [54]. Sequences with more than 30% gaps are subsequently removed from the alignment. We then excluded alignment columns that contained 50% or more gaps from model inference and subsequent couplings predictions. Lastly, sequences were clustered at 80% sequence identity and then downweighted according to the cluster size to reduce redundancy. This resulted in an alignment of 5663 unique sequences with an effective number of 1080.4 non-redundant sequences (sequences/alignment length = 4.8) included in model inference and coupling prediction. The coupling scores of residue pairs were further normalized by estimating the background noise analogously to the procedure described in Hopf et al., 2014 [28]. Evaluation of the co-evolution prediction was done in the light of topology predictions obtained from deltaG, resulting in four predicted TM segments: (7, 38), (50, 75), (163, 193), (194, 211). Python (Python Software Foundation, http://www.python.org) and Ipython/Jupyter notebooks [55] were used for data analysis. The multiple sequence alignment, EC scores file, a contact map of the strongest couplings and an Ipython notebook of the analysis are available as supplement (S3 Table, S8 Fig, S2 and S3 Files).

### Electron microscopy and image analysis

Isolated SpaPR^FLAG^ complexes were deposited on glow-discharged carbon coated copper-palladium grids and stained with 0.75% uranyl formate. Micrograph acquisition was performed on a FEI Tecnai F30 Polara at 300 kV, equipped with a Gatan Ultrascan 4000 UHS CCD (4k x 4k pixels, physical pixel size of 15 μm), using the LEGINON automated image acquisition system [56]. The corrected magnification was 71950x, resulting in a pixel size of 2.08 Å/pixel. 11202 particles were picked from the micrographs with EMAN2 boxer [57]. Particle images were first subjected to a maximum-likelihood classification and alignment (ML2D) in XMIPP [58] and then further processed in IMAGIC-5 (Image Science Software GmbH) through multi-reference alignment and classification by multi-variate statistical analysis.

### SpaPR pore assessment by biotin maleimide labeling

SpaP or SpaPQR^FLAG^ were moderately overexpressed in *S*. Typhimurium strain SB1770 (Δ*prgHIJK*, *flhD*::*tet*) from a rhamnose-inducible medium copy number plasmid by induction with 20 μM rhamnose. BM labeling was performed essentially as previously described [29], with minor modifications: After 3 h of induction, 0.2 ODU of bacterial cells were transferred to a fresh reaction tube and brought to the same volume by addition of fresh LB broth. Cells were labeled by addition of BM (EZ-link maleimide-PEG2-biotin, Thermo Pierce, final concentration 0.4 mM) for 30 min at room temperature with gentle agitation. The reaction was quenched by addition 2M β-mercaptoethanol to a final concentration of 10 mM. Cells were pelleted, re-suspended in SB buffer and incubated at 70°C for 10 min. BM labeling of proteins was analyzed by SDS PAGE, Western blotting, and detection of BM with streptavidin DyLight 800 dye (Thermo pierce). Scanning of the PVDF membrane and image analysis was performed with a Li-Cor Odyssey system and image Studio 2.1.10 (Li-Cor).

For subcellular fractionation, BM-labeled bacterial cells were pelleted by centrifugation. The culture supernatant was harvested and TCA precipitated. The bacterial cell pellet was resuspended and used to prepare the periplasmic and cytoplasmic fractions as described elsewhere. Briefly, pellets were resuspended by pipetting gently in ice-cold spheroplast buffer (40% sucrose, 33 mM Tris-HCl, pH 8.0) with freshly prepared lysozyme to a final concentration of 200 μg/ml, 50 μg/ml DNAse and 1.5 mM EDTA. The mixture was left on ice for 30 min with gentle stirring. Spheroplasts were stabilized by adding 20 mM MgCl_2_ and centrifuged at 3000 x g for 10 min at 4°C. The supernatant was transferred to ultracentrifugation tubes and centrifuged at 30 krpm for 30 min at 4°C in a Beckman TLA55 rotor to remove insoluble material. The supernatant (periplasmic fraction) was collected into fresh tube. The cytoplasmic fraction was prepared by resuspending the pellet of spheroplasts in 1 ml of 20 mM Tris-HCl, pH 8.0 and subsequent lysis by bead milling as described above. Lysates were transferred to ultracentrifugation tubes and centrifuged at 55 krpm for 45 min at 4°C in a Beckman TLA55 rotor. The supernatant (cytoplasmic fraction) was collected into fresh tubes.

## Supporting Information

S1 Table Raw data of the size exclusion chromatography-multi angle laser light scattering analysis of the purified SpaPR complex. (XLSX)

S2 Table Crosslinked adducts identified by mass spectrometry. (XLSX)

S3 Table EC scores of coupling prediction. (XLSX)

S4 Table Strains and plasmids. (XLSX)

S5 Table Oligonucleotides. (XLSX)

S1 Fig Three detector calibration of the SEC-MALLS equipment and error calculation. (PDF)

S2 Fig Functional analysis of SpaP and SpaR *p*Bpa mutants. (A) Type III dependent secretion into the culture supernatant of indicated *p*Bpa mutants of SpaP and SpaR, respectively, was assayed by SDS PAGE and immunodetection of the early substrate InvJ and the intermediate substrate SipB. For two of the secretion-deficient SpaP mutants (T15X, M187X), assembly of SpaP into the needle complex was confirmed by 2-dimensional blue native/SDS PAGE (Fig 3C). Further, many secretion-defective *p*Bpa mutants showed productive crosslinks to other needle complex components. These results suggest that secretion-deficiency was not due to gross structural defects but rather the result of subtle conformational changes. (B) As in (A) but detailing secretion profiles of chromosome-encoded SpaP-*p*Bpa mutants.
(TIF)

S3 Fig SDS PAGE analysis of isolated needle complexes of SpaP and SpaR *p*Bpa mutants with and without UV photocrosslinking for mass spectrometrical identification of crosslinking partners. (A) Immunodetection of SpaP^FLAG^ and SpaR^FLAG^, respectively, on Western blots of purified needle complexes of *S*. Typhimurium expressing indicated SpaP or SpaR *p*Bpa mutants separated by SDS PAGE. Each sample is shown with and without UV-irradiation to induce photocrosslinking of the *p*Bpa to neighboring interaction partners. Identified interaction partners are indicated at the respective bands. A summary of the MS identifications is shown in S2 Table. (B) Coomassie stained SDS PAGE gels of the UV-irradiated samples shown in (A). Gel pieces were cut out at positions of crosslinking adducts identified by Western blotting and immunodetection for subsequent in gel Trypsin digestion and MS analysis.
(TIF)

S4 Fig Class averages of negative-stained isolated SpaPR complexes imaged by electron microscopy. 91 classes are shown. The length of the scale bar in the upper left corner represents 50 Å.
(TIF)

S5 Fig Controls for general bacterial lysis for biotin maleimide labeling experiments. (A) Coomassie-stained gel (left) and immunodetection (cytoplasmic marker RNA polymerase (RNApol), periplasmic marker maltose binding protein (MBP), right) of SDS PAGE-separated whole cell lysates and cell culture supernatants, respectively, of *S*. Typhimurium Δ*prgHIJK*, *flhD*::*tet* moderately overexpressing indicated proteins from a medium copy number plasmid (pT12). Equal culture volumes were loaded in each well. (B) Immunodetection of SDS PAGE-separated cell culture supernatants, periplasmic fractions, and cytoplasmic fractions, respectively, of *S*. Typhimurium Δ*prgHIJK*, *flhD*::*tet* moderately overexpressing indicated proteins from a medium copy number plasmid (pT12). Equal culture volumes were loaded in each well. RNApol serves as a marker protein for cytoplasmic proteins, MBP serves as a marker protein for periplasmic proteins. (C) Growth curves of *S*. Typhimurium Δ*prgHIJK*, *flhD*::*tet* overexpressing indicated proteins from a medium copy number plasmid (pT12) with the indicated concentrations of rhamnose.
(TIF)

S6 Fig Prediction of topology and of the propensity of membrane integration of SpaR. (A) Topcons prediction of SpaR (topcons.cbr.su.se). (B) Prediction of ΔG for membrane integration propensity of SpaR using a sliding window between 18 and 31 amino acids (dgpred.cbr.su.se). (C) Protter visualization of the topology model of SpaR comprising 3 TM helices and an N-out/C-in orientation. Positions of detected crosslinks of SpaR to other T3SS components are indicated in color.
(TIF)

S7 Fig Position and sequence of epitope tags used in SpaP, SpaR, and SpaS. (PDF)

S8 Fig Contact map of top 291 residue couplings. Abbreviations: norm. normalized, exp. experimentally.
(PNG)

S1 File SEC-MALLS ASTRA calculations. (PDF)

S2 File Input alignment in fasta format. (A2M)

S3 File Notebook containing couplings analysis. (HTML)
