# Supplementary material for: Structural and Functional Characterization of the Bacterial Type III Secretion Export Apparatus
Source: PLoS Pathog. 2016 Dec 15;12(12):e1006071. doi: 10.1371/journal.ppat.1006071 (PMC5158082; doi:10.1371/journal.ppat.1006071)
Supplement: S1 Fig — (PDF) [file ppat.1006071.s006.pdf]

## Three detector calibration

Column: S200#5

"three det" calibration constant= 1.96E-01

**dn/dc polypeptide** 0.175

**dn/dc sugars** 0.134

**MW=k*(LS)*(UV)/(A*(RI^2))**

where k is a system constant (from calibration)

A=extinction coefficient expressed in terms of weight concnetration [mL/(mg*cm-1)]

see: Hayashi, Matsui and Takagi
Methods in Enzymology ((1989) Vol 172, 514-528

Based on analyses of standard proteins
run in the same buffer
from Hayashi, Matsui and Takagi

| | |
|---|---|
| Error UV | 0.005 |
| Error LS | 0.005 |
| Error RI | 0.005 |

**Three det. Approach**

| protein | (LS)*(UV)/(A*(RI^2)) ratio | MW [kDa] | computed | error |
|---|---|---|---|---|
| OVA(1) | 239.7 | 43 | 47 | 9% |
| BAM | 1074.9 | 220 | 211 | 4% |
| CA | 164.7 | 29 | 32 | 12% |
| AD | 807.030 | 149 | 159 | 6% |
| BSA (1) | 341.2 | 66 | 67 | 2% |
| | | | AVERAGE | 7% |

**k2 constant calculations**

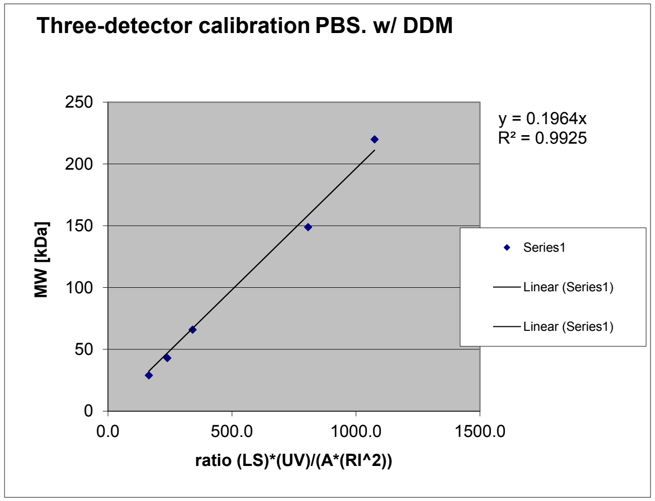


Fig S1
