# Supplementary figures and images for: Structural and Functional Characterization of the Bacterial Type III Secretion Export Apparatus
Source: PLoS Pathog. 2016 Dec 15;12(12):e1006071. doi: 10.1371/journal.ppat.1006071 (PMC5158082; doi:10.1371/journal.ppat.1006071)

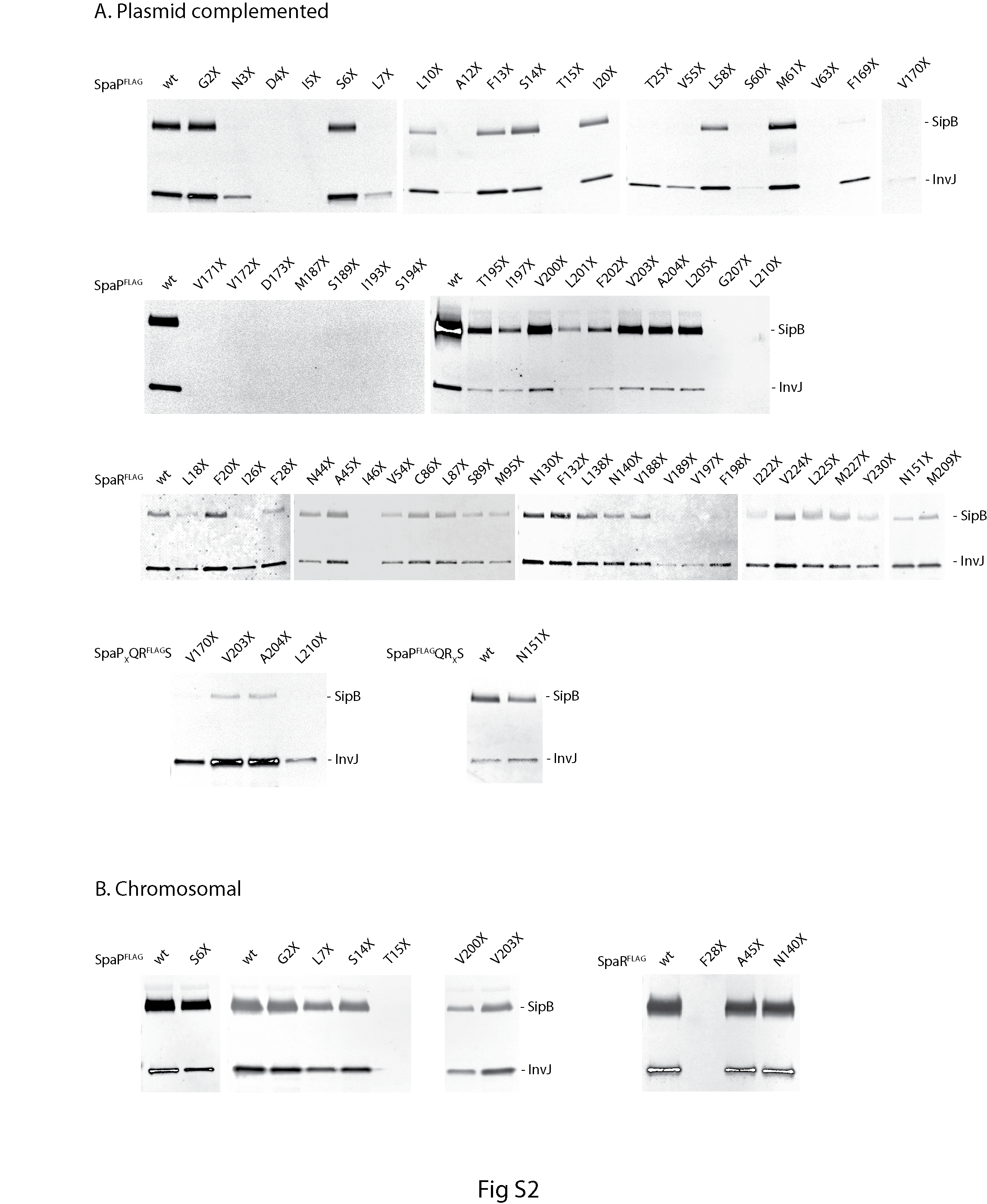

Supplement: S2 Fig — (A) Type III dependent secretion into the culture supernatant of indicated pBpa mutants of SpaP and SpaR, respectively, was assayed by SDS PAGE and immunodetection of the early substrate InvJ and the intermediate substrate SipB. For two of the secretion-deficient SpaP mutants (T15X, M187X), assembly of SpaP into the needle complex was confirmed by 2-dimensional blue native/SDS PAGE (Fig 3C). Further, many secretion-defective pBpa mutants showed productive crosslinks to other needle complex components. These results suggest that secretion-deficiency was not due to gross structural defects but rather the result of subtle conformational changes. (B) As in (A) but detailing secretion profiles of chromosome-encoded SpaP-pBpa mutants. (TIF) [file ppat.1006071.s007.tif]

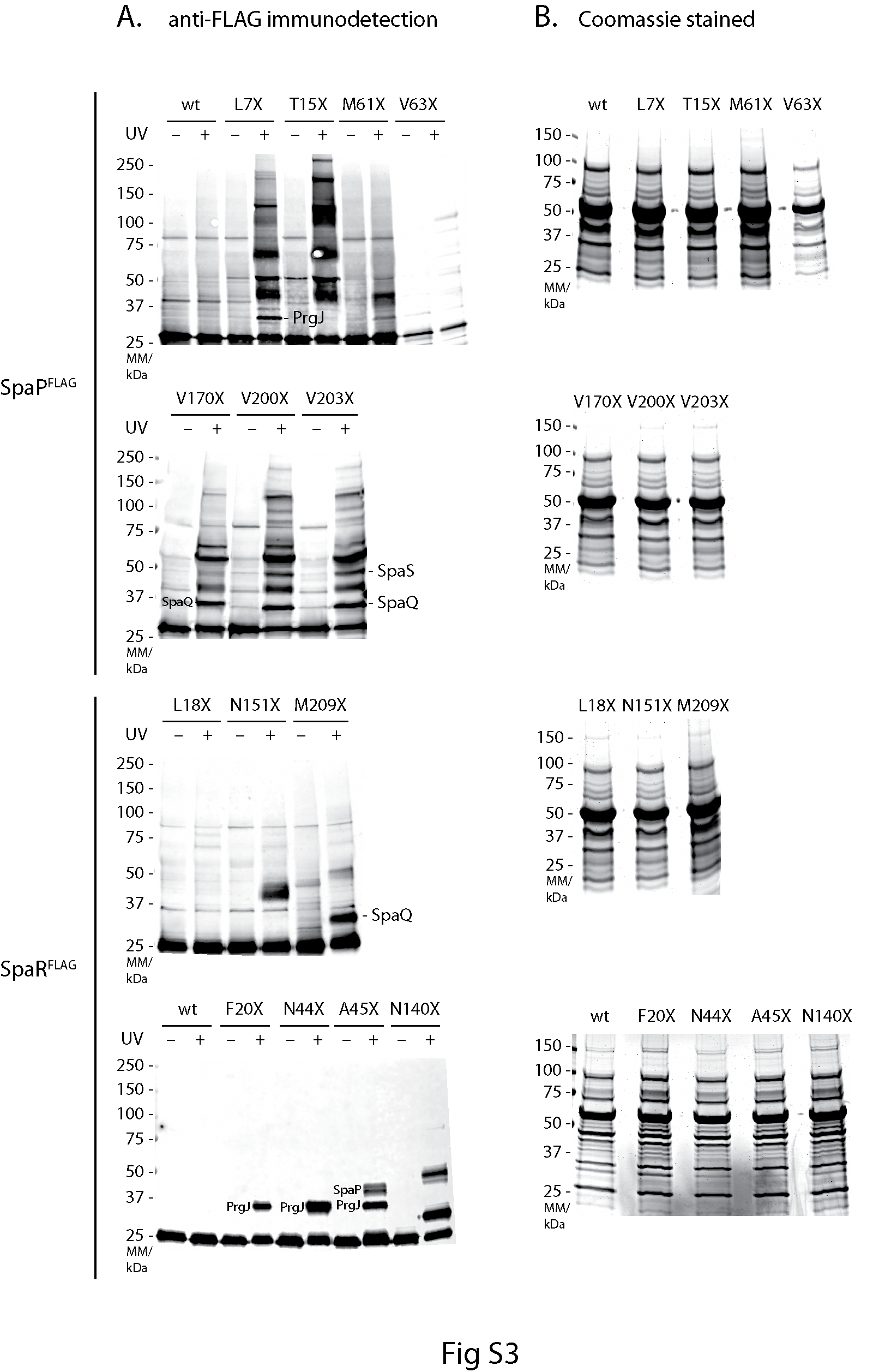

Supplement: S3 Fig — (A) Immunodetection of SpaPFLAG and SpaRFLAG, respectively, on Western blots of purified needle complexes of S. Typhimurium expressing indicated SpaP or SpaR pBpa mutants separated by SDS PAGE. Each sample is shown with and without UV-irradiation to induce photocrosslinking of the pBpa to neighboring interaction partners. Identified interaction partners are indicated at the respective bands. A summary of the MS identifications is shown in S2 Table. (B) Coomassie stained SDS PAGE gels of the UV-irradiated samples shown in (A). Gel pieces were cut out at positions of crosslinking adducts identified by Western blotting and immunodetection for subsequent in gel Trypsin digestion and MS analysis. (TIF) [file ppat.1006071.s008.tif]

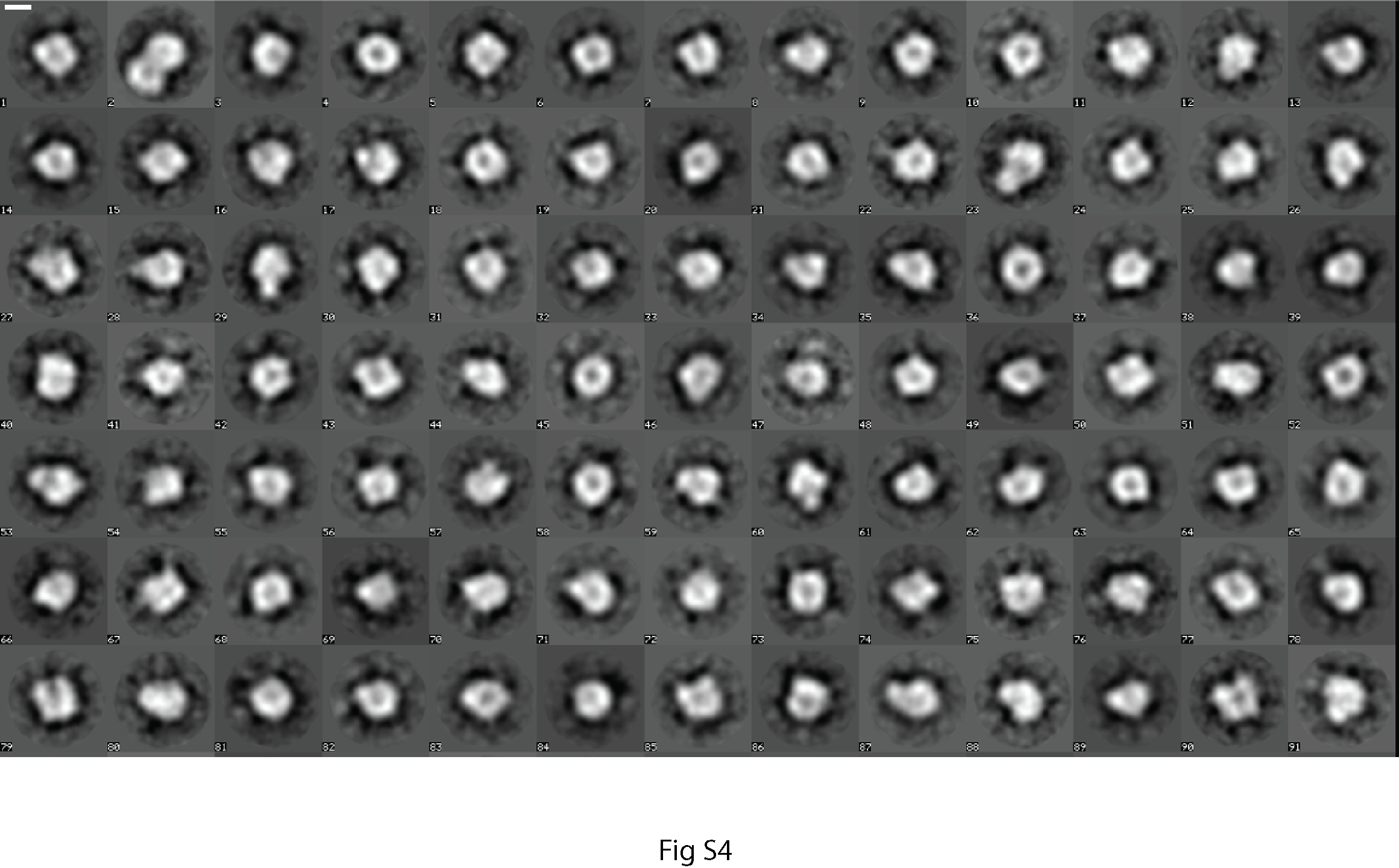

Supplement: S4 Fig — 91 classes are shown. The length of the scale bar in the upper left corner represents 50 Å. (TIF) [file ppat.1006071.s009.tif]

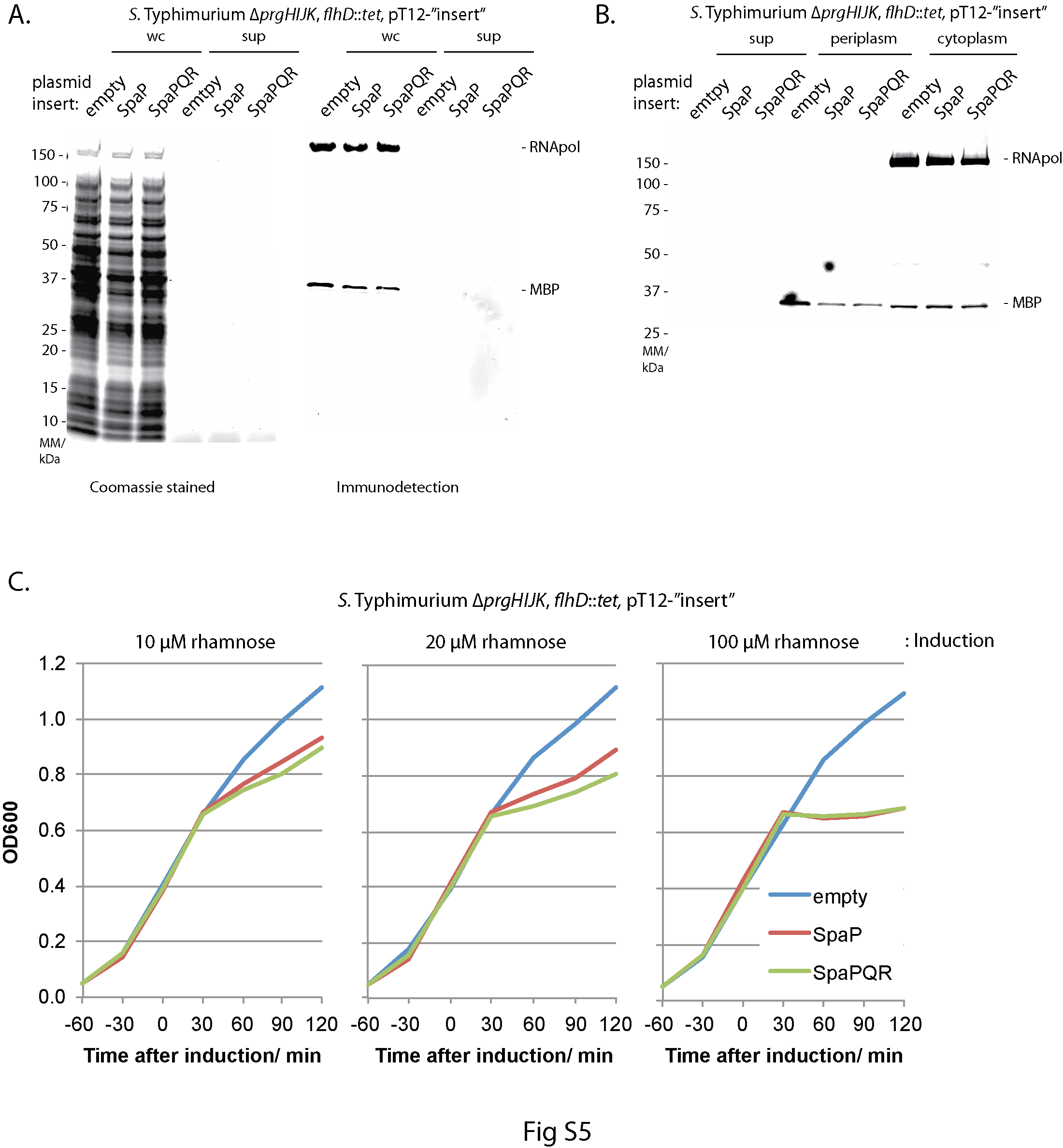

Supplement: S5 Fig — (A) Coomassie-stained gel (left) and immunodetection (cytoplasmic marker RNA polymerase (RNApol), periplasmic marker maltose binding protein (MBP), right) of SDS PAGE-separated whole cell lysates and cell culture supernatants, respectively, of S. Typhimurium ΔprgHIJK, flhD::tet moderately overexpressing indicated proteins from a medium copy number plasmid (pT12). Equal culture volumes were loaded in each well. (B) Immunodetection of SDS PAGE-separated cell culture supernatants, periplasmic fractions, and cytoplasmic fractions, respectively, of S. Typhimurium ΔprgHIJK, flhD::tet moderately overexpressing indicated proteins from a medium copy number plasmid (pT12). Equal culture volumes were loaded in each well. RNApol serves as a marker protein for cytoplasmic proteins, MBP serves as a marker protein for periplasmic proteins. (C) Growth curves of S. Typhimurium ΔprgHIJK, flhD::tet overexpressing indicated proteins from a medium copy number plasmid (pT12) with the indicated concentrations of rhamnose. (TIF) [file ppat.1006071.s010.tif]

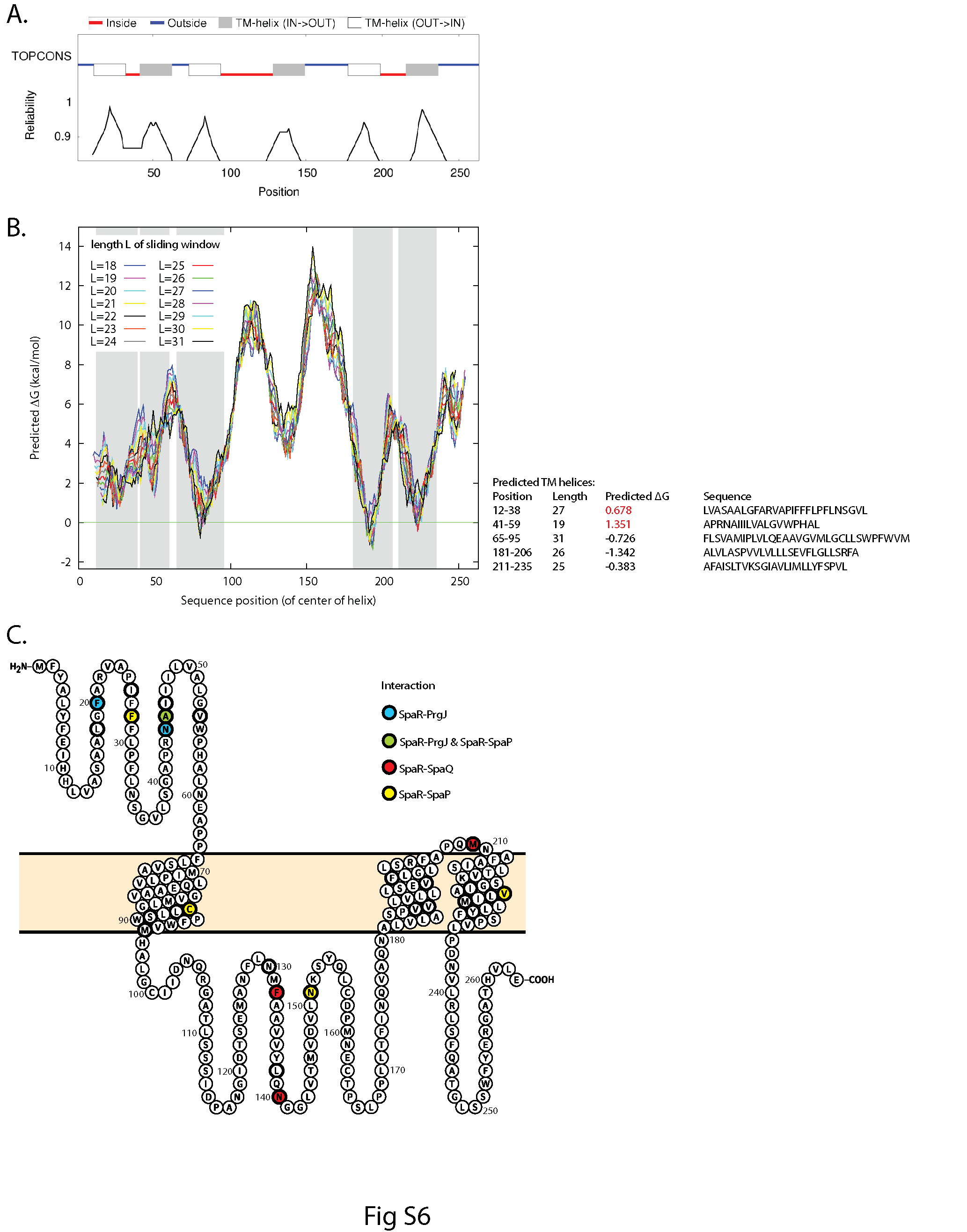

Supplement: S6 Fig — (A) Topcons prediction of SpaR (topcons.cbr.su.se). (B) Prediction of ΔG for membrane integration propensity of SpaR using a sliding window between 18 and 31 amino acids (dgpred.cbr.su.se). (C) Protter visualization of the topology model of SpaR comprising 3 TM helices and an N-out/C-in orientation. Positions of detected crosslinks of SpaR to other T3SS components are indicated in color. (TIF) [file ppat.1006071.s011.tif]

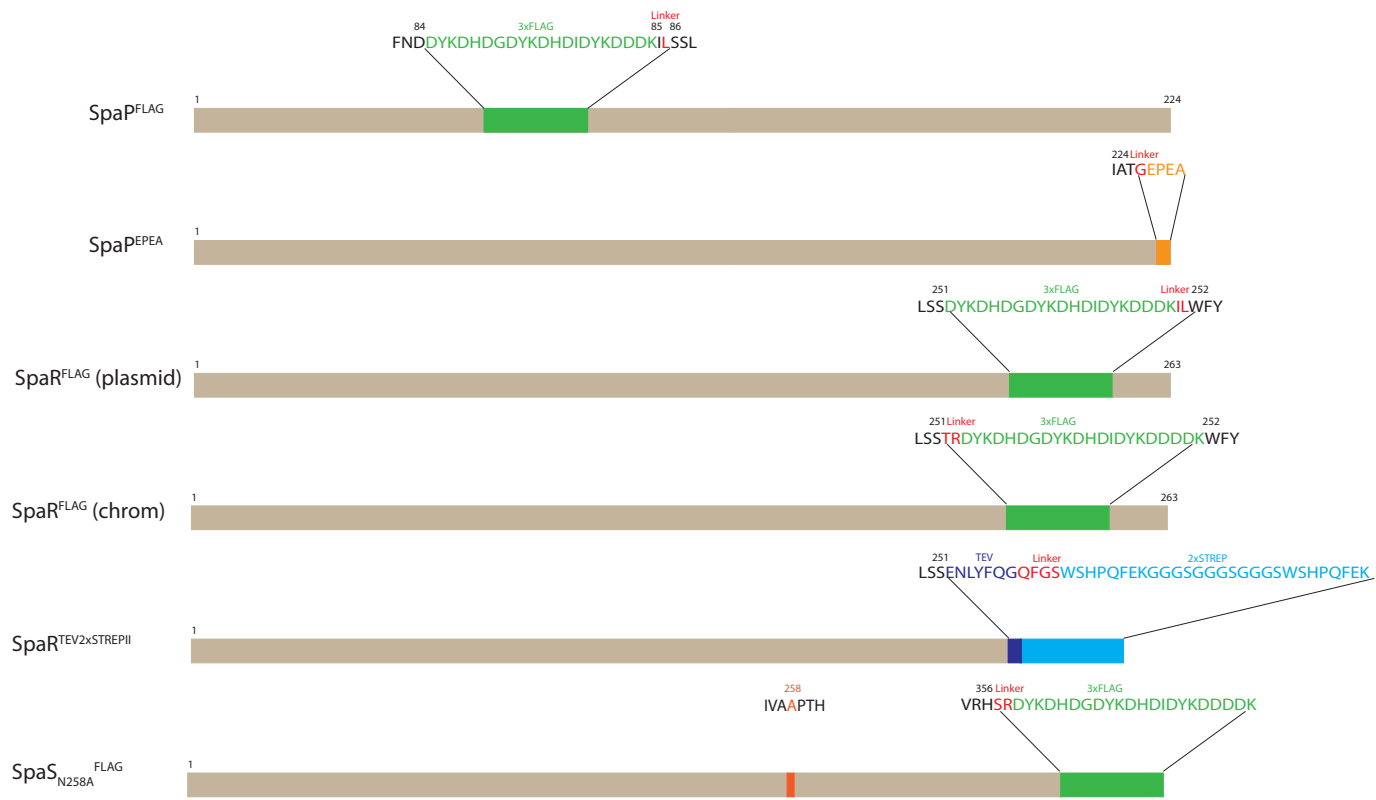

Fig S7

Supplement: S7 Fig — (PDF) [file ppat.1006071.s012.pdf]

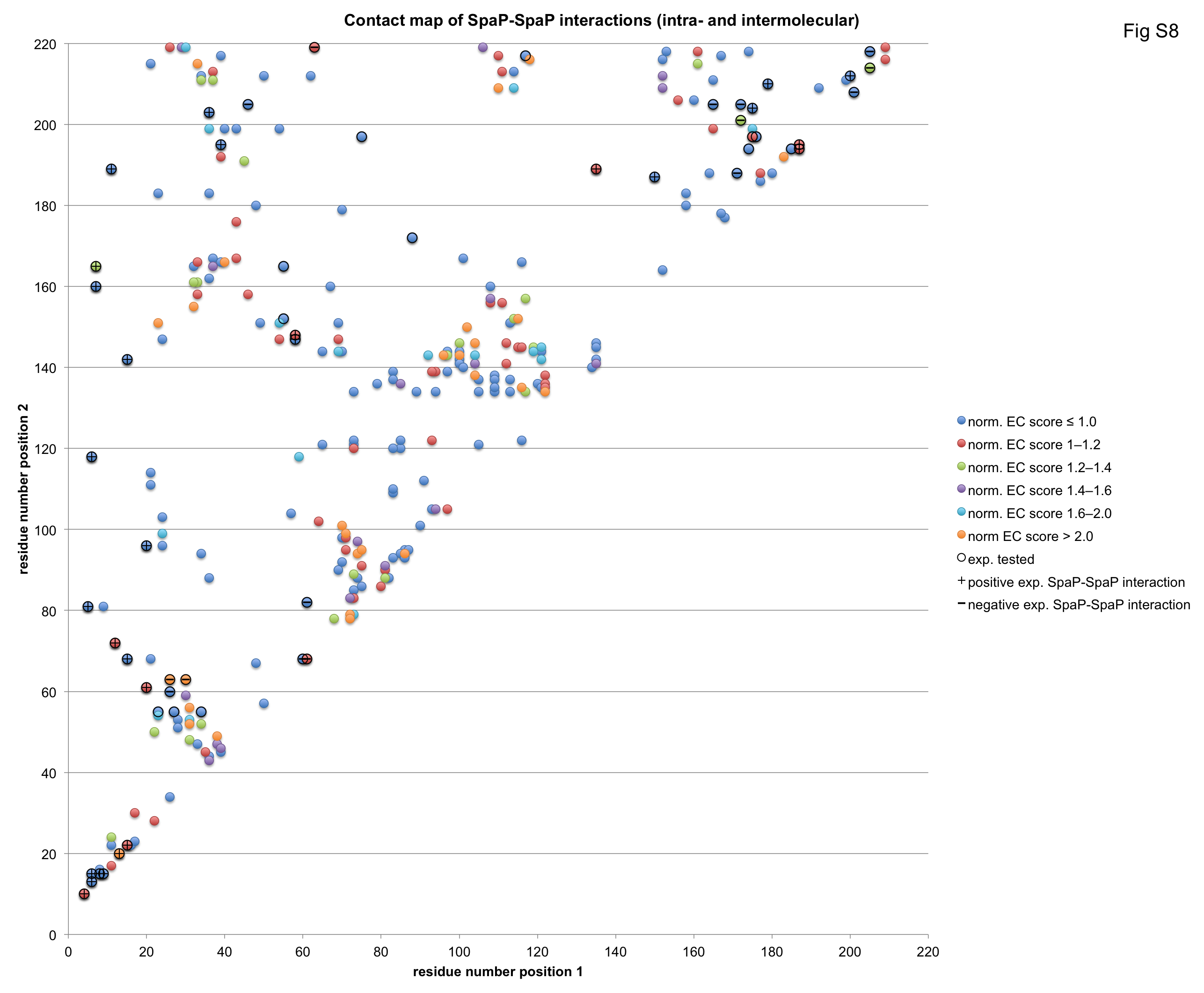

Supplement: S8 Fig — Abbreviations: norm. normalized, exp. experimentally. (PNG) [file ppat.1006071.s013.png]
