# Supplementary material for: Structural and Functional Characterization of the Bacterial Type III Secretion Export Apparatus
Source: PLoS Pathog. 2016 Dec 15;12(12):e1006071. doi: 10.1371/journal.ppat.1006071 (PMC5158082; doi:10.1371/journal.ppat.1006071)
Supplement: S1 File — (PDF) [file ppat.1006071.s014.pdf]

ASTRA 5.3.4 Summary Report for PR_550ul[Sam_032211a]_P_N_complex

Experiment name: C:\Documents and Settings\ef55.YALE.000\Desktop\Mar_11\Sam\PR_550ul[Sam_032211a]_P_N_complex
Sample: PR_550ul (S200#5)
Processing Operator: Folta-Stogniew, Ewa
Collection Operator: Folta-Stogniew, Ewa
Collection Astra Version: 5.3.4.10

CONFIGURATION

Light scattering instrument: DAWN HELEOS
- **Cell type:** K5
- **Laser wavelength:** 658.0 nm
- **Calibration constant:** 4.7560e-5 1/(V cm)
- **Replaced detector:** 12
- **Temperature control:** n/a
- **Temperature:** n/a

RI Instrument: Optilab rEX
UV Instrument: Generic UV instrument
QELS instrument:
- **Model:** Wyatt QELS+
- **Use temperature probe:** yes

Solvent: PBS, Aqueous
- **Refractive index:** 1.340
- **Viscosity:** 8.9450e-3 g/(cm sec) (valid if QELS temperature not used)

Flow rate: 0.500 mL/min

PROCESSING

Processing time: Thursday March 24, 2011 03:24 PM Eastern Daylight Time
Collection time: Tuesday March 22, 2011 06:34 PM Eastern Daylight Time
QELS delay time range: 1.00e-6 to 1.00 sec
QELS threshold values: 1.0 to 300.0 nm
Detectors used: 4 5 6 7 8 9 10 11 13 14 15 16 17 18
Concentration detector: RI
Mass results fitting: none (fit degree: n/a)
Radius results fitting: none (fit degree: n/a)

	Peak 1
Peak limits (mL)	11.187-11.924
dn/dc (mL/g)	0.154
A_2 (mol mL/g²)	0.000
UV ext. (mL/(g cm))	0.000
Model	Debye
Fit degree	1
Injected mass (g)	0.0000
Calc. mass (g)	1.0146e-4

RESULTS

	Peak 1
**Polydispersity**	
Mw/Mn	1.000(0.1%)
Mz/Mn	1.000(0.1%)
**Molar mass moments (g/mol)**	
Mn	3.109e+5(0.0%)
Mp	3.117e+5(0.0%)
Mv	n/a
Mw	3.109e+5(0.0%)
Mz	3.109e+5(0.1%)

ASTRA 5.3.4 Summary Report for PR_550ul[Sam_032211a]_P_N_pp

Experiment name: C:\Documents and Settings\ef55.YALE.000\Desktop\Mar_11\Sam\PR_550ul[Sam_032211a]_P_N_pp
Sample: PR_550ul (S200#5)
Processing Operator: Folta-Stogniew, Ewa
Collection Operator: Folta-Stogniew, Ewa
Collection Astra Version: 5.3.4.10

CONFIGURATION

Light scattering instrument: DAWN HELEOS
- **Cell type:** K5
- **Laser wavelength:** 658.0 nm
- **Calibration constant:** 4.7560e-5 1/(V cm)
- **Replaced detector:** 12
- **Temperature control:** n/a
- **Temperature:** n/a

RI Instrument: Optilab rEX
UV Instrument: Generic UV instrument
QELS instrument:
- **Model:** Wyatt QELS+
- **Use temperature probe:** yes

Solvent: PBS, Aqueous
- **Refractive index:** 1.340
- **Viscosity:** 8.9450e-3 g/(cm sec) (valid if QELS temperature not used)

Flow rate: 0.500 mL/min

PROCESSING

Processing time: Thursday March 24, 2011 03:24 PM Eastern Daylight Time
Collection time: Tuesday March 22, 2011 06:34 PM Eastern Daylight Time
QELS delay time range: 1.00e-6 to 1.00 sec
QELS threshold values: 1.0 to 300.0 nm
Detectors used: 4 5 6 7 8 9 10 11 13 14 15 16 17 18
Concentration detector: RI
Mass results fitting: none (fit degree: n/a)
Radius results fitting: none (fit degree: n/a)

	Peak 1
**Peak limits (mL)**	11.187-11.924
**dn/dc (mL/g)**	0.300
**A_2 (mol mL/g²)**	0.000
**UV ext. (mL/(g cm))**	0.000
**Model**	Debye
**Fit degree**	1
**Injected mass (g)**	0.0000
**Calc. mass (g)**	5.2085e-5

RESULTS

	Peak 1
**Polydispersity**	
**Mw/Mn**	1.000(0.1%)
**Mz/Mn**	1.000(0.1%)
**Molar mass moments (g/mol)**	
**Mn**	1.596e+5(0.0%)
**Mp**	1.600e+5(0.0%)
**Mv**	n/a
**Mw**	1.596e+5(0.0%)
**Mz**	1.596e+5(0.1%)
