# Supplementary material for: Structural and Functional Characterization of the Bacterial Type III Secretion Export Apparatus
Source: PLoS Pathog. 2016 Dec 15;12(12):e1006071. doi: 10.1371/journal.ppat.1006071 (PMC5158082; doi:10.1371/journal.ppat.1006071)
Supplement: S3 File — (HTML) [file ppat.1006071.s016.html]

SpaP\_analysis


In [1]:

```
import IPython.core.display as di

# This line will hide code by default when the notebook is exported as HTML
di.display_html('<script>jQuery(function() {if (jQuery("body.notebook_app").length == 0) { jQuery(".input_area").toggle(); jQuery(".prompt").toggle();}});</script>', raw=True)

# This line will add a button to toggle visibility of code blocks, for use with the HTML export version
di.display_html('''<button onclick="jQuery('.input_area').toggle(); jQuery('.prompt').toggle();">Toggle code</button>''', raw=True)
```

Toggle code

# Coevolution analysis for SPAP\_SALTY¶

In [2]:

```
%matplotlib inline
```

In [3]:

```
%load_ext autoreload
%autoreload 2
```

In [4]:

```
import mpld3
from mpld3 import plugins
import matplotlib.pyplot as plt
import seaborn as sns
import numpy as np
import pandas as pd
from IPython.display import Image
from operator import itemgetter
from itertools import groupby
from collections import OrderedDict
from handle_sequence_files import read_fasta_to_list
```

```
/Users/schaerfe/Documents/Privat/Dropbox/anaconda/lib/python2.7/site-packages/matplotlib/__init__.py:872: UserWarning: axes.color_cycle is deprecated and replaced with axes.prop_cycle; please use the latter.
  warnings.warn(self.msg_depr % (key, alt_key))
```

In [5]:

```
pd.set_option('display.max_rows', 500)
```

# Functions¶

In [6]:

```
def read_monomer_contacts(ECfile, filter_close=True, sort_score=True, tm=False, filter_tm=False):
    """
    reads an EC with options to filter i->i+5 residues and sort by EC score
    """
    if tm:
        couplings_raw = preprocess_TM_ECs(ECfile, filter=filter_tm)
        col_names = ['i', 'res_i', 'j', 'res_j', 'EC', 'tm_flag']
        couplings = pd.DataFrame(couplings_raw, columns=col_names)
        couplings = couplings[['i', 'res_i', 'j', 'res_j', 'tm_flag', 'EC']]
    else:
        col_names = ['i', 'res_i', 'j', 'res_j', 'skip', 'EC']
        couplings = pd.read_csv(ECfile, sep=' ', header=None, names=col_names)

    # remove the i to i+5 contacts
    if filter_close:
        couplings = couplings.loc[abs(couplings['i'] - couplings['j']) > 5]
    if sort_score:
        couplings.sort_values(by=['EC'], ascending=False, inplace=True)
    return couplings

def plot_ec_distibution(ecs, show_confidence=True, column="CN", ax=None, figsize=(10, 10), color='teal', label=''):
    """ plots distribution of ECs scores and shows noise threshold """
    if not ax:
        fig, ax = plt.subplots(1, 1, figsize=figsize)
    ecs.hist(column, bins=100, color=color, alpha=0.3, lw=0, grid=False, ax=ax, label=label)
    confidence_threshold = np.abs(ecs[column].min())
    if show_confidence:
        plt.axvline(x=confidence_threshold, ymin = 0, color='grey', linestyle='dashed')

        
def add_normalized_score(ecs, score_col='EC'):
    """ normalize score by noise level """
    ecs_added = ecs.copy()
    min_ec = np.abs(ecs_added[score_col].min())
    ecs_added["normalized_score"] = ecs_added.apply((lambda x: x[score_col]/min_ec), axis=1)
    return ecs_added

def label_col(aa1, res1, aa2, res2, score):
    """ convert EC row into one label """
    return "{}{}, {}{} ({:.2f})".format(aa1, res1, aa2, res2, score)

html_temp = '<div style="background-color:rgba(255,255,255,0.75);"><b>&nbsp;{}&nbsp;</b></div>'
    
def add_label(ecs):
    """ add columns with label string """
    return_ecs = ecs.copy()
    return_ecs["label1"] = return_ecs.apply(lambda x: label_col(x.res_i, x.i, x.res_j, x.j, x.EC), axis=1)
    return_ecs["label2"] = return_ecs.apply(lambda x: label_col(x.res_j, x.j, x.res_i, x.i, x.EC), axis=1)
    return return_ecs

def plot_ECs(plotting_ecs, fig=None, tm_segments=None, interactive=True, figsize=(6,6),
             color_col=None, size_col=None):
    """
    create scatter plot of couplings. Filter couplings dataframe beforehand
    to only show a certain number of ECs.
    """
    from matplotlib.patches import Rectangle
    ecs = plotting_ecs.copy()
    
    if not fig:
        fig, ax = plt.subplots(1, 1, figsize=figsize)
    
    else:
        ax = plt.gca()
    
    if color_col is None:
        ecs["color"] = 'orange'
        color_col = 'color'
    if size_col is None:
        ecs["size"] = 20
        size_col = 'size'

    ax_ec1 = plt.scatter(ecs.i, ecs.j, marker='o', color=ecs[color_col], s=ecs[size_col], lw=0)
    ax_ec2 = plt.scatter(ecs.j, ecs.i, marker='o', color=ecs[color_col], s=ecs[size_col], lw=0)
    
    # adjust axis limits
    y_lim = ax.get_ylim()
    ax.set_ylim(0, max(y_lim))
    ax.set_xlim(0, max(y_lim))
    
    if tm_segments:
        
        for (start, end) in tm_segments:
            ax.add_patch(Rectangle((0, start), max(y_lim), end - start, facecolor="lightgrey", alpha=0.3))
            ax.add_patch(Rectangle((start, 0), end - start, max(y_lim), facecolor="lightgrey", alpha=0.3))

    ax.invert_yaxis()
    
    if interactive:
        if not "label1" in ecs.columns:
            ecs = add_label(ecs)
        labels_html1 = [html_temp.format(l) for l in ecs.label1]
        labels_html2 = [html_temp.format(l) for l in ecs.label2]
        plugins.connect(fig, plugins.PointHTMLTooltip(ax_ec1, labels_html2, voffset=-35, hoffset=-5))
        plugins.connect(fig, plugins.PointHTMLTooltip(ax_ec2, labels_html1, voffset=-35, hoffset=-5))
        return fig
    
def plot_contactmap(xtal_contacts_list, plotting_ecs, annotations=[], distance_threshold=6.0, fig=None,
                    tm_segments=None, interactive=True, figsize=(6,6),
                    color_col=None, size_col=None):
    """
    Plot a protein contact map and ECs.
    Uses a precalculated dataframe for all residue pair distances and a
    customizable threshold to define what residues are considered "in-contact"
    in the structure.
    """
    from matplotlib.patches import Rectangle
    ecs = plotting_ecs.copy()
    
    if not fig:
        fig, ax = plt.subplots(1, 1, figsize=figsize)
    
    else:
        ax = plt.gca()
    
    # plot close residues
    for xtal_contacts in xtal_contacts_list:
        xtal_close = xtal_contacts.loc[(xtal_contacts.distance <= distance_threshold)]
        ax_xtal1 = plt.scatter(xtal_close.res1_uniprot, xtal_close.res2_uniprot, marker='o', 
                               color='lightgrey', s=60, lw=0)
        ax_xtal2 = plt.scatter(xtal_close.res2_uniprot, xtal_close.res1_uniprot, marker='o',
                               color='lightgrey', s=60, lw=0)
    
    # plot transmembrane segments
    max_i = max(ecs.i.tolist() + ecs.j.tolist())
    y_lim = (0, max_i)
    if tm_segments:
        for (start, end) in tm_segments:
            ax.add_patch(Rectangle((0, start), max(y_lim), end - start,
                                   facecolor="lightgrey", edgecolor='none', alpha=.3, zorder=0))
            ax.add_patch(Rectangle((start, 0), end - start, max(y_lim),
                                   facecolor="lightgrey", edgecolor='none', alpha=.3, zorder=0))
        
    # plot ECs
    if color_col is None:
        ecs["color"] = 'orange'
        color_col = 'color'
    if size_col is None:
        ecs["size"] = 20
        size_col = 'size'
    ax_ec1 = plt.scatter(ecs.i, ecs.j, marker='o', color=ecs[color_col], s=ecs[size_col], lw=0)
    ax_ec2 = plt.scatter(ecs.j, ecs.i, marker='o', color=ecs[color_col], s=ecs[size_col], lw=0)
    
    # adjust axis limits
    ax.set_ylim(0, max_i)
    ax.set_xlim(0, max_i)
    y_lim = ax.get_ylim()
    ax.invert_yaxis()
    
    # add missing ranges, if available
    for (annot_start, annot_end) in annotations:
        start_frac = float(annot_start)/max_i
        end_frac = float(annot_end)/max_i
        if (end_frac - start_frac) < 0.01:
            continue
        if start_frac > 1.0:
            continue
        if end_frac > 1.0:
            end_frac = 1.0
        ax.annotate('', xy=(start_frac, 1.01), xycoords='axes fraction', xytext=(end_frac, 1.01), 
                    arrowprops=dict(arrowstyle="-", color="#b71224", linewidth=2))
        ax.annotate('', xy=(1.01, 1 - start_frac), xycoords='axes fraction', xytext=(1.01, 1 - end_frac), 
                    arrowprops=dict(arrowstyle="-", color="#b71224", linewidth=2))
        ax.annotate('', xy=(start_frac, -0.01), xycoords='axes fraction', xytext=(end_frac, -0.01), 
                    arrowprops=dict(arrowstyle="-", color="#b71224", linewidth=2))
        ax.annotate('', xy=(-0.01, 1 - start_frac), xycoords='axes fraction', xytext=(-0.01, 1 - end_frac), 
                    arrowprops=dict(arrowstyle="-", color="#b71224", linewidth=2))
    
    if interactive:
        if not "label1" in ecs.columns:
            ecs = add_label(ecs)
        labels_html1 = [html_temp.format(l) for l in ecs.label1]
        labels_html2 = [html_temp.format(l) for l in ecs.label2]
        plugins.connect(fig, plugins.PointHTMLTooltip(ax_ec1, labels_html2, voffset=-35, hoffset=-5))
        plugins.connect(fig, plugins.PointHTMLTooltip(ax_ec2, labels_html1, voffset=-35, hoffset=-5))
    return fig


def percentage_gaps(msa_list):
    """ Get gap percentage for each column in alignment."""
    gaps = [".", "-"]
    gap_count = []
    seqs = np.array(map(list, msa_list[1:]))  # ignore query sequence
    n_rows, n_cols = seqs.shape
    for c in range(n_cols):
        g = 0
        for gc in gaps:
            col = seqs[:, c]
            g_x = (col == gc).sum()

            if g_x > 0:
                g += g_x
        g = g / float(n_rows)
        gap_count.append(g)
    return gap_count


def draw_alignment_characteristics(sequences):
    """Plot % gaps per position in alignment."""
    data = percentage_gaps(sequences)
    label = 'Percentage gaps in column'

    fig, ax = plt.subplots(figsize=(8, 5))
    ax.plot(range(len(data)), data, "-")

    ax.set_xlim(-1, len(sequences[0]))
    ax.set_ylabel(label, fontsize='large')
    ax.set_xlabel('Residue index', fontsize='large')

    ax.tick_params(axis='x', pad=5)
    ax.tick_params(axis='y', pad=15)
    # ax.set_ylim(0, 1)

    ax.axhline(y=0.5, xmin=-1, xmax=len(sequences[0]), color='#676767', ls="--", alpha=0.5)


def calculate_ec_enrichment(ec_df, rank_threshold=None, ij_cols=['i', 'j'], score_col='EC'):
    """
    Calculate cumulative EC strength on residues (a.k.a "EC enrichment") as
    defined in 2012 membrane paper (Hopf et al, 2012, Cell). Uses all ECs in ec_list up to
    threshold X (e.g, 1 * protein length). If EC list is prefiltered, set rank_threshold to None.

    code written bei TAH and CPS.
    """
    # average coupling strength of all ECs in list
    if rank_threshold is not None:
        ec_df.sort_values(by=[score_col], ascending=False, inplace=True)
        ec_df = ec_df[:rank_threshold]

    mean_strength = ec_df[score_col].mean()

    # calculate the weighted and unweighted degree of each node i and j in network
    ij = pd.melt(ec_df, id_vars=[score_col], value_vars=ij_cols, value_name='residue')
    enrichment = ij.groupby('residue').agg({score_col: OrderedDict([('count', 'count'),
                                                                    ('mean_score', 'mean'),
                                                                    ('median_score', 'median'),
                                                                    ('summed_score', sum)])})
    enrichment[('EC', "normalized_strength")] = enrichment[('EC', 'summed_score')] / mean_strength

    return mean_strength, enrichment
```

# Load data¶

In [7]:

```
tm_spap = [(7, 38), (50, 75), (163, 193), (194, 211)]
```

# Analysis¶

## Alignment features¶

In [8]:

```
spap_monomer_alignment = "./SPAP_SALTY_hmmer_plm_n5_m50_f70_t0.2_r1-224_id100_e3.a2m"
```

In [9]:

```
alignment = read_fasta_to_list(spap_monomer_alignment)
ids, sequences = zip(*alignment)
```

In [10]:

```
len(sequences)
```

```
/Users/schaerfe/Documents/Privat/Dropbox/anaconda/lib/python2.7/site-packages/IPython/core/formatters.py:92: DeprecationWarning: DisplayFormatter._ipython_display_formatter_default is deprecated: use @default decorator instead.
  def _ipython_display_formatter_default(self):
/Users/schaerfe/Documents/Privat/Dropbox/anaconda/lib/python2.7/site-packages/IPython/core/formatters.py:669: DeprecationWarning: PlainTextFormatter._singleton_printers_default is deprecated: use @default decorator instead.
  def _singleton_printers_default(self):
```

Out[10]:

```
7034
```

### Plot gaps per column in alignment¶

In [11]:

```
with sns.axes_style("white"):
    draw_alignment_characteristics(sequences)
```

### Extract regions in alignment that were excluded from model inference¶

In [12]:

```
query = sequences[0]
```

In [13]:

```
excluded = [i+1 for i, c in enumerate(query) if c.islower()]
```

In [14]:

```
exclude_ranges = []
for k, g in groupby(enumerate(excluded), lambda (i,x):i-x):
    current_range = map(itemgetter(1), g)
    exclude_ranges.append((min(current_range), max(current_range)))
```

In [15]:

```
print "Excluded ranges", ", ".join(map(str, exclude_ranges))
```

```
Excluded ranges (1, 3), (76, 77), (123, 133), (220, 224)
```

## SPAP couplings as predicted by monomer prediction¶

### Read ECs¶

In [16]:

```
spap_monomer_ecs = "./SPAP_SALTY_hmmer_plm_n5_m50_f70_t0.2_r1-224_id100_e3_ECs.txt"
```

In [17]:

```
spap_monomer = read_monomer_contacts(spap_monomer_ecs, filter_close=True)
```

In [18]:

```
del spap_monomer["skip"]
```

In [19]:

```
spap_monomer = add_normalized_score(spap_monomer)
len(spap_monomer.loc[spap_monomer.normalized_score >= 0.8])
```

Out[19]:

```
291
```

### Write ECs with headings and normalized score for supplement¶

In [20]:

```
spap_monomer.rename(columns=dict(i="position1", res_i="residue_1", j="position2", res_j="residue_2",
                                 EC="EC_score", normalized_score="normalized_EC_score"))\
            .to_csv("./SPAP_SALTY_hmmer_plm_n5_m50_f70_t0.2_r1-224_id100_e3_Couplings.txt",
                    index=False, sep="\t", float_format='%.3f')
```

### Make Figure for supplement¶

In [21]:

```
mon = spap_monomer.loc[spap_monomer.normalized_score >= 0.8]
```

In [22]:

```
with sns.axes_style("white"):
    fig = plot_contactmap([], mon, annotations=[], tm_segments=tm_spap, interactive=False, figsize=(8, 8))
    plt.savefig("contact_map_top291.pdf", dpi=300)
```

### Annotate segment of residues excluded from prediction with red bar¶

In [23]:

```
with sns.axes_style("white"):
    fig = plot_contactmap([], mon, annotations=exclude_ranges, tm_segments=tm_spap, interactive=False, figsize=(8, 8))
```

This is contact map shows the first 291 contacts of SPAP as predicted by using the alignment of this protein alone. The rank threshold was determined using the normalized couplings score as defined in the EVcomplex paper. Grey boxes are the transmembrane segments and the darker grey shape is the 2D representation of the models of the SPAP periplasmic domain. The overlap between dark grey and orange thus shows that the evolutionary record agrees well with the likely structure in the periplasmic region.

In [24]:

```
mon
```

Out[24]:

|  | i | res\_i | j | res\_j | EC | normalized\_score |
| --- | --- | --- | --- | --- | --- | --- |
| 11348 | 71 | A | 99 | Y | 1.161020 | 6.444025 |
| 14210 | 96 | L | 143 | I | 0.826015 | 4.584642 |
| 11462 | 72 | Y | 79 | D | 0.806505 | 4.476356 |
| 4959 | 30 | F | 63 | V | 0.624113 | 3.464023 |
| 11742 | 74 | Y | 94 | E | 0.591960 | 3.285564 |
| 15065 | 104 | I | 138 | I | 0.576967 | 3.202348 |
| 13007 | 86 | S | 94 | E | 0.568822 | 3.157140 |
| 6754 | 40 | A | 166 | Y | 0.546043 | 3.030710 |
| 11215 | 70 | D | 101 | D | 0.513989 | 2.852800 |
| 6317 | 38 | R | 49 | N | 0.503746 | 2.795948 |
| 14648 | 100 | R | 143 | I | 0.498092 | 2.764567 |
| 5387 | 32 | I | 155 | I | 0.496718 | 2.756941 |
| 5123 | 31 | S | 52 | L | 0.496567 | 2.756103 |
| 16494 | 118 | A | 216 | I | 0.495293 | 2.749031 |
| 16157 | 115 | F | 152 | L | 0.489485 | 2.716795 |
| 4249 | 26 | C | 63 | V | 0.485157 | 2.692773 |
| 15739 | 110 | E | 209 | T | 0.466477 | 2.589094 |
| 5620 | 33 | V | 215 | L | 0.458170 | 2.542987 |
| 1788 | 13 | F | 20 | I | 0.450929 | 2.502797 |
| 3781 | 23 | S | 151 | A | 0.445250 | 2.471277 |
| 15073 | 104 | I | 146 | L | 0.438003 | 2.431054 |
| 11461 | 72 | Y | 78 | E | 0.433749 | 2.407443 |
| 11874 | 75 | F | 95 | G | 0.422156 | 2.343098 |
| 16762 | 122 | R | 134 | D | 0.421808 | 2.341167 |
| 16232 | 116 | E | 135 | K | 0.409165 | 2.270994 |
| 14868 | 102 | Y | 150 | Y | 0.399168 | 2.215508 |
| 19845 | 183 | L | 192 | T | 0.391576 | 2.173370 |
| 5127 | 31 | S | 56 | A | 0.385929 | 2.142027 |
| 6117 | 36 | M | 199 | L | 0.358025 | 1.987151 |
| 3922 | 24 | G | 99 | Y | 0.345695 | 1.918716 |
| 16511 | 119 | Q | 144 | F | 0.344319 | 1.911078 |
| 16687 | 121 | K | 145 | A | 0.343814 | 1.908276 |
| 5124 | 31 | S | 53 | N | 0.339069 | 1.881939 |
| 11595 | 73 | V | 79 | D | 0.334785 | 1.858162 |
| 5102 | 30 | F | 219 | Y | 0.333569 | 1.851413 |
| 11111 | 69 | H | 144 | F | 0.330469 | 1.834207 |
| 15070 | 104 | I | 143 | I | 0.324114 | 1.798934 |
| 19536 | 175 | V | 199 | L | 0.323711 | 1.796698 |
| 16684 | 121 | K | 142 | S | 0.322983 | 1.792657 |
| 3697 | 23 | S | 54 | G | 0.313963 | 1.742593 |
| 8958 | 54 | G | 151 | A | 0.302404 | 1.678437 |
| 13756 | 92 | V | 143 | I | 0.301523 | 1.673547 |
| 16121 | 114 | F | 209 | T | 0.295880 | 1.642227 |
| 9681 | 59 | L | 118 | A | 0.289602 | 1.607382 |
| 11745 | 74 | Y | 97 | D | 0.287560 | 1.596048 |
| 15490 | 108 | D | 157 | S | 0.286339 | 1.589271 |
| 16938 | 135 | K | 141 | P | 0.284709 | 1.580224 |
| 6315 | 38 | R | 47 | P | 0.283632 | 1.574247 |
| 18281 | 152 | L | 209 | T | 0.282666 | 1.568885 |
| 5974 | 36 | M | 43 | L | 0.275982 | 1.531787 |
| 11466 | 72 | Y | 83 | N | 0.274394 | 1.522973 |
| 4926 | 29 | K | 219 | Y | 0.273075 | 1.515652 |
| 18284 | 152 | L | 212 | S | 0.272535 | 1.512655 |
| 15351 | 106 | Y | 219 | Y | 0.271982 | 1.509585 |
| 6481 | 39 | N | 46 | I | 0.269734 | 1.497108 |
| 6252 | 37 | V | 165 | L | 0.269030 | 1.493201 |
| 13958 | 94 | E | 105 | K | 0.268488 | 1.490193 |
| 4955 | 30 | F | 59 | L | 0.268106 | 1.488072 |
| 12384 | 81 | T | 91 | H | 0.267677 | 1.485691 |
| 12916 | 85 | I | 136 | D | 0.261043 | 1.448871 |
| 15068 | 104 | I | 141 | P | 0.258263 | 1.433441 |
| 20406 | 205 | L | 214 | G | 0.250935 | 1.392768 |
| 7594 | 45 | Q | 191 | V | 0.244418 | 1.356597 |
| 16064 | 114 | F | 152 | L | 0.241049 | 1.337898 |
| 18845 | 161 | I | 215 | L | 0.240220 | 1.333296 |
| 3510 | 22 | A | 50 | M | 0.240131 | 1.332802 |
| 16512 | 119 | Q | 145 | A | 0.240094 | 1.332597 |
| 12381 | 81 | T | 88 | L | 0.237910 | 1.320475 |
| 5566 | 33 | V | 161 | I | 0.234864 | 1.303569 |
| 5788 | 34 | F | 211 | L | 0.233525 | 1.296137 |
| 5642 | 34 | F | 52 | L | 0.233146 | 1.294033 |
| 5393 | 32 | I | 161 | I | 0.232629 | 1.291164 |
| 16322 | 117 | N | 134 | D | 0.231181 | 1.283127 |
| 14651 | 100 | R | 146 | L | 0.230564 | 1.279703 |
| 1405 | 11 | L | 24 | G | 0.225744 | 1.252950 |
| 5119 | 31 | S | 48 | S | 0.224356 | 1.245246 |
| 14321 | 97 | D | 143 | I | 0.223361 | 1.239724 |
| 16345 | 117 | N | 157 | S | 0.220037 | 1.221274 |
| 6298 | 37 | V | 211 | L | 0.219532 | 1.218471 |
| 19403 | 172 | V | 201 | L | 0.218942 | 1.215197 |
| 747 | 7 | L | 165 | L | 0.217058 | 1.204740 |
| 10919 | 68 | M | 78 | E | 0.216483 | 1.201549 |
| 11605 | 73 | V | 89 | S | 0.216403 | 1.201105 |
| 6614 | 39 | N | 192 | T | 0.215201 | 1.194433 |
| 18848 | 161 | I | 218 | Q | 0.215097 | 1.193856 |
| 20457 | 209 | T | 219 | Y | 0.213905 | 1.187240 |
| 7247 | 43 | L | 167 | L | 0.212369 | 1.178715 |
| 14294 | 97 | D | 105 | K | 0.208697 | 1.158334 |
| 19982 | 187 | M | 195 | T | 0.208147 | 1.155281 |
| 10385 | 64 | M | 102 | Y | 0.207812 | 1.153422 |
| 9553 | 58 | L | 148 | P | 0.207594 | 1.152212 |
| 15489 | 108 | D | 156 | K | 0.206858 | 1.148127 |
| 11599 | 73 | V | 83 | N | 0.205032 | 1.137992 |
| 19981 | 187 | M | 194 | S | 0.204818 | 1.136804 |
| 11870 | 75 | F | 91 | H | 0.204441 | 1.134712 |
| 16766 | 122 | R | 138 | I | 0.203151 | 1.127552 |
| 15869 | 112 | V | 146 | L | 0.199737 | 1.108603 |
| 15864 | 112 | V | 141 | P | 0.197679 | 1.097180 |
| 15783 | 111 | L | 156 | K | 0.197490 | 1.096131 |
| 4392 | 26 | C | 219 | Y | 0.196359 | 1.089854 |
| 11347 | 71 | A | 98 | G | 0.196353 | 1.089821 |
| 19534 | 175 | V | 197 | I | 0.196343 | 1.089765 |
| 2173 | 15 | T | 22 | A | 0.196235 | 1.089166 |
| 5571 | 33 | V | 166 | Y | 0.196119 | 1.088522 |
| 6300 | 37 | V | 213 | K | 0.195791 | 1.086701 |
| 11344 | 71 | A | 95 | G | 0.195423 | 1.084659 |
| 16764 | 122 | R | 136 | D | 0.195304 | 1.083998 |
| 5563 | 33 | V | 158 | A | 0.195055 | 1.082616 |
| 3152 | 20 | I | 61 | M | 0.194858 | 1.081523 |
| 18536 | 156 | K | 206 | D | 0.194834 | 1.081390 |
| 10349 | 63 | V | 219 | Y | 0.194721 | 1.080763 |
| 11114 | 69 | H | 147 | L | 0.194358 | 1.078748 |
| 19610 | 177 | S | 188 | M | 0.193345 | 1.073125 |
| 5806 | 35 | V | 45 | Q | 0.193341 | 1.073103 |
| 2560 | 17 | L | 30 | F | 0.192342 | 1.067558 |
| 7721 | 46 | I | 158 | A | 0.192327 | 1.067475 |
| 8954 | 54 | G | 147 | L | 0.191327 | 1.061925 |
| 15747 | 110 | E | 217 | L | 0.191320 | 1.061886 |
| 19051 | 165 | L | 199 | L | 0.190714 | 1.058523 |
| 13981 | 94 | E | 139 | E | 0.189880 | 1.053894 |
| 1398 | 11 | L | 17 | L | 0.188907 | 1.048493 |
| 11636 | 73 | V | 120 | L | 0.188448 | 1.045945 |
| 12252 | 80 | V | 86 | S | 0.188432 | 1.045857 |
| 3488 | 22 | A | 28 | V | 0.188245 | 1.044819 |
| 7256 | 43 | L | 176 | V | 0.188088 | 1.043947 |
| 16986 | 135 | K | 189 | S | 0.188034 | 1.043648 |
| 16150 | 115 | F | 145 | A | 0.187904 | 1.042926 |
| 5 | 4 | D | 10 | L | 0.187853 | 1.042643 |
| 20454 | 209 | T | 216 | I | 0.186758 | 1.036565 |
| 12383 | 81 | T | 90 | K | 0.185508 | 1.029628 |
| 15840 | 111 | L | 213 | K | 0.185115 | 1.027446 |
| 16763 | 122 | R | 135 | K | 0.184668 | 1.024965 |
| 16242 | 116 | E | 145 | A | 0.184112 | 1.021879 |
| 9924 | 61 | M | 68 | M | 0.183681 | 1.019487 |
| 13867 | 93 | D | 139 | E | 0.183639 | 1.019254 |
| 1647 | 12 | A | 72 | Y | 0.183522 | 1.018605 |
| 13861 | 93 | D | 122 | R | 0.181650 | 1.008214 |
| 19407 | 172 | V | 205 | L | 0.180083 | 0.999517 |
| 12911 | 85 | I | 120 | L | 0.180024 | 0.999190 |
| 16853 | 134 | D | 140 | K | 0.179640 | 0.997058 |
| 12637 | 83 | N | 93 | D | 0.178325 | 0.989760 |
| 2363 | 16 | L | 22 | A | 0.178214 | 0.989144 |
| 13315 | 88 | L | 172 | V | 0.177645 | 0.985985 |
| 18288 | 152 | L | 216 | I | 0.177504 | 0.985203 |
| 4596 | 28 | V | 53 | N | 0.177204 | 0.983538 |
| 14647 | 100 | R | 142 | S | 0.177131 | 0.983133 |
| 1557 | 11 | L | 189 | S | 0.176752 | 0.981029 |
| 4594 | 28 | V | 51 | T | 0.176578 | 0.980063 |
| 7279 | 43 | L | 199 | L | 0.176448 | 0.979342 |
| 3185 | 20 | I | 96 | L | 0.176352 | 0.978809 |
| 16591 | 120 | L | 136 | D | 0.176321 | 0.978637 |
| 13976 | 94 | E | 134 | D | 0.176274 | 0.978376 |
| 3344 | 21 | I | 68 | M | 0.176130 | 0.977577 |
| 5397 | 32 | I | 165 | L | 0.175474 | 0.973936 |
| 19057 | 165 | L | 205 | L | 0.174458 | 0.968297 |
| 19916 | 185 | M | 194 | S | 0.173912 | 0.965266 |
| 3919 | 24 | G | 96 | L | 0.173759 | 0.964417 |
| 6787 | 40 | A | 199 | L | 0.173087 | 0.960687 |
| 19713 | 179 | V | 210 | L | 0.172262 | 0.956108 |
| 10557 | 65 | W | 144 | F | 0.171977 | 0.954526 |
| 1069 | 9 | A | 81 | T | 0.171269 | 0.950597 |
| 16939 | 135 | K | 142 | S | 0.171093 | 0.949620 |
| 7768 | 46 | I | 205 | L | 0.170768 | 0.947816 |
| 15162 | 105 | K | 121 | K | 0.169696 | 0.941866 |
| 13008 | 86 | S | 95 | G | 0.169677 | 0.941761 |
| 18236 | 152 | L | 164 | Y | 0.169433 | 0.940406 |
| 14317 | 97 | D | 139 | E | 0.169072 | 0.938403 |
| 2553 | 17 | L | 23 | S | 0.168994 | 0.937970 |
| 5975 | 36 | M | 44 | Q | 0.168784 | 0.936804 |
| 19185 | 168 | P | 177 | S | 0.168569 | 0.935611 |
| 5789 | 34 | F | 212 | S | 0.167699 | 0.930782 |
| 3813 | 23 | S | 183 | L | 0.166717 | 0.925332 |
| 20324 | 200 | V | 212 | S | 0.166602 | 0.924693 |
| 16230 | 116 | E | 122 | R | 0.166408 | 0.923617 |
| 14779 | 101 | D | 167 | L | 0.165972 | 0.921197 |
| 742 | 7 | L | 160 | K | 0.165604 | 0.919154 |
| 3385 | 21 | I | 111 | L | 0.165526 | 0.918721 |
| 3959 | 24 | G | 147 | L | 0.165275 | 0.917328 |
| 3698 | 23 | S | 55 | V | 0.165174 | 0.916767 |
| 6617 | 39 | N | 195 | T | 0.165030 | 0.915968 |
| 15952 | 113 | Q | 134 | D | 0.164788 | 0.914625 |
| 10545 | 65 | W | 121 | K | 0.164458 | 0.912793 |
| 4246 | 26 | C | 60 | S | 0.164368 | 0.912294 |
| 6101 | 36 | M | 183 | L | 0.163928 | 0.909852 |
| 19063 | 165 | L | 211 | L | 0.163500 | 0.907476 |
| 19174 | 167 | L | 217 | L | 0.163351 | 0.906649 |
| 16686 | 121 | K | 144 | F | 0.163139 | 0.905473 |
| 20338 | 201 | L | 208 | W | 0.162912 | 0.904213 |
| 16677 | 121 | K | 135 | K | 0.162890 | 0.904091 |
| 18633 | 158 | A | 180 | L | 0.162881 | 0.904041 |
| 20304 | 199 | L | 211 | L | 0.162804 | 0.903613 |
| 14646 | 100 | R | 141 | P | 0.162638 | 0.902692 |
| 2280 | 15 | T | 142 | S | 0.162595 | 0.902453 |
| 18778 | 160 | K | 206 | D | 0.162398 | 0.901360 |
| 8060 | 48 | S | 180 | L | 0.162383 | 0.901277 |
| 11965 | 75 | F | 197 | I | 0.161953 | 0.898890 |
| 9552 | 58 | L | 147 | L | 0.161361 | 0.895604 |
| 808 | 8 | I | 15 | T | 0.161360 | 0.895599 |
| 14752 | 101 | D | 140 | K | 0.161216 | 0.894799 |
| 12885 | 85 | I | 94 | E | 0.160994 | 0.893567 |
| 11601 | 73 | V | 85 | I | 0.160978 | 0.893478 |
| 5682 | 34 | F | 94 | E | 0.160849 | 0.892762 |
| 19343 | 171 | V | 188 | M | 0.160824 | 0.892624 |
| 12913 | 85 | I | 122 | R | 0.160625 | 0.891519 |
| 6080 | 36 | M | 162 | G | 0.160390 | 0.890215 |
| 5645 | 34 | F | 55 | V | 0.160193 | 0.889121 |
| 12672 | 83 | N | 139 | E | 0.160064 | 0.888405 |
| 6017 | 36 | M | 88 | L | 0.159959 | 0.887823 |
| 1403 | 11 | L | 22 | A | 0.159747 | 0.886646 |
| 16943 | 135 | K | 146 | L | 0.159680 | 0.886274 |
| 16125 | 114 | F | 213 | K | 0.159360 | 0.884498 |
| 11247 | 70 | D | 144 | F | 0.158904 | 0.881967 |
| 8263 | 50 | M | 57 | L | 0.158740 | 0.881057 |
| 10199 | 62 | F | 212 | S | 0.158485 | 0.879641 |
| 16263 | 116 | E | 166 | Y | 0.158379 | 0.879053 |
| 7960 | 48 | S | 67 | I | 0.158336 | 0.878814 |
| 5465 | 33 | V | 47 | P | 0.158163 | 0.877854 |
| 15570 | 109 | R | 138 | I | 0.157522 | 0.874296 |
| 9110 | 55 | V | 152 | L | 0.157164 | 0.872309 |
| 16405 | 117 | N | 217 | L | 0.156615 | 0.869262 |
| 20410 | 205 | L | 218 | Q | 0.156563 | 0.868974 |
| 13006 | 86 | S | 93 | D | 0.156455 | 0.868374 |
| 409 | 6 | S | 13 | F | 0.156132 | 0.866582 |
| 13844 | 93 | D | 105 | K | 0.156056 | 0.866160 |
| 3388 | 21 | I | 114 | F | 0.155446 | 0.862774 |
| 411 | 6 | S | 15 | T | 0.155288 | 0.861897 |
| 6254 | 37 | V | 167 | L | 0.154876 | 0.859610 |
| 13396 | 89 | S | 134 | D | 0.154769 | 0.859016 |
| 11068 | 69 | H | 90 | K | 0.154726 | 0.858778 |
| 10852 | 67 | I | 160 | K | 0.154594 | 0.858045 |
| 6121 | 36 | M | 203 | V | 0.154192 | 0.855814 |
| 11865 | 75 | F | 86 | S | 0.154171 | 0.855697 |
| 6480 | 39 | N | 45 | Q | 0.153965 | 0.854554 |
| 11637 | 73 | V | 121 | K | 0.153666 | 0.852894 |
| 18124 | 150 | Y | 187 | M | 0.153569 | 0.852356 |
| 4420 | 27 | F | 55 | V | 0.153474 | 0.851829 |
| 8405 | 50 | M | 212 | S | 0.153354 | 0.851163 |
| 15569 | 109 | R | 137 | E | 0.153168 | 0.850130 |
| 19608 | 177 | S | 186 | M | 0.153107 | 0.849792 |
| 13492 | 90 | K | 101 | D | 0.152992 | 0.849154 |
| 15955 | 113 | Q | 137 | E | 0.152947 | 0.848904 |
| 275 | 5 | I | 81 | T | 0.152793 | 0.848049 |
| 1005 | 9 | A | 15 | T | 0.152176 | 0.844625 |
| 15566 | 109 | R | 134 | D | 0.151706 | 0.842016 |
| 11206 | 70 | D | 92 | V | 0.151598 | 0.841416 |
| 13620 | 91 | H | 112 | V | 0.151571 | 0.841267 |
| 19577 | 176 | V | 197 | I | 0.151500 | 0.840873 |
| 12507 | 82 | F | 88 | L | 0.151202 | 0.839219 |
| 19511 | 174 | L | 218 | Q | 0.151189 | 0.839146 |
| 15969 | 113 | Q | 151 | A | 0.151077 | 0.838525 |
| 9779 | 60 | S | 68 | M | 0.150938 | 0.837753 |
| 3926 | 24 | G | 103 | L | 0.150765 | 0.836793 |
| 14322 | 97 | D | 144 | F | 0.150588 | 0.835811 |
| 19730 | 180 | L | 188 | M | 0.150313 | 0.834284 |
| 11118 | 69 | H | 151 | A | 0.150239 | 0.833874 |
| 13129 | 87 | S | 95 | G | 0.150141 | 0.833330 |
| 15567 | 109 | R | 135 | K | 0.149813 | 0.831509 |
| 9372 | 57 | L | 104 | I | 0.149657 | 0.830643 |
| 11638 | 73 | V | 122 | R | 0.149531 | 0.829944 |
| 9123 | 55 | V | 165 | L | 0.149438 | 0.829428 |
| 8188 | 49 | N | 151 | A | 0.149218 | 0.828207 |
| 15493 | 108 | D | 160 | K | 0.149179 | 0.827990 |
| 16942 | 135 | K | 145 | A | 0.149066 | 0.827363 |
| 15167 | 105 | K | 137 | E | 0.149030 | 0.827163 |
| 12163 | 79 | D | 136 | D | 0.149026 | 0.827141 |
| 6588 | 39 | N | 166 | Y | 0.148911 | 0.826503 |
| 3478 | 21 | I | 215 | L | 0.148711 | 0.825393 |
| 20141 | 192 | T | 209 | T | 0.148630 | 0.824943 |
| 19541 | 175 | V | 204 | A | 0.148624 | 0.824910 |
| 11736 | 74 | Y | 88 | L | 0.148318 | 0.823211 |
| 2219 | 15 | T | 68 | M | 0.148215 | 0.822640 |
| 11212 | 70 | D | 98 | G | 0.148172 | 0.822401 |
| 9006 | 54 | G | 199 | L | 0.147963 | 0.821241 |
| 4220 | 26 | C | 34 | F | 0.147908 | 0.820936 |
| 6639 | 39 | N | 217 | L | 0.147712 | 0.819848 |
| 18356 | 153 | S | 218 | Q | 0.147146 | 0.816706 |
| 15164 | 105 | K | 134 | D | 0.147125 | 0.816590 |
| 809 | 8 | I | 16 | L | 0.147038 | 0.816107 |
| 18986 | 164 | Y | 188 | M | 0.146732 | 0.814409 |
| 512 | 6 | S | 118 | A | 0.146505 | 0.813149 |
| 12653 | 83 | N | 109 | R | 0.146157 | 0.811217 |
| 12654 | 83 | N | 110 | E | 0.146000 | 0.810346 |
| 19487 | 174 | L | 194 | S | 0.145882 | 0.809691 |
| 12670 | 83 | N | 137 | E | 0.145705 | 0.808708 |
| 12664 | 83 | N | 120 | L | 0.145538 | 0.807782 |
| 11639 | 73 | V | 134 | D | 0.145422 | 0.807138 |
| 9936 | 61 | M | 82 | F | 0.145328 | 0.806616 |
| 19135 | 167 | L | 178 | S | 0.145312 | 0.806527 |
| 14649 | 100 | R | 144 | F | 0.145079 | 0.805234 |
| 11282 | 70 | D | 179 | V | 0.145068 | 0.805173 |
| 18636 | 158 | A | 183 | L | 0.144563 | 0.802370 |

## Strongly coupled residues¶

In [25]:

```
top_L_spap = len(mon)
mean_score_spap, enrichment_spap = calculate_ec_enrichment(mon)
print 'Average coupling score:', mean_score_spap, '(in top', top_L_spap, 'ECs).'
```

```
Average coupling score: 0.222716058419 (in top 291 ECs).
```

In [26]:

```
enrichment_spap.sort_values(by=[('EC', 'count'), ('EC', 'normalized_strength')], ascending=False).head(20)
```

Out[26]:

|  | EC | | | | |
| --- | --- | --- | --- | --- | --- |
|  | count | mean\_score | median\_score | summed\_score | normalized\_strength |
| residue |  |  |  |  |  |
| 135 | 9 | 0.206569 | 0.171093 | 1.859118 | 8.347481 |
| 134 | 9 | 0.196968 | 0.164788 | 1.772713 | 7.959520 |
| 122 | 8 | 0.207893 | 0.183159 | 1.663145 | 7.467558 |
| 73 | 8 | 0.194283 | 0.174713 | 1.554265 | 6.978684 |
| 83 | 8 | 0.175152 | 0.153110 | 1.401215 | 6.291486 |
| 94 | 7 | 0.302467 | 0.189880 | 2.117267 | 9.506575 |
| 152 | 7 | 0.255691 | 0.241049 | 1.789836 | 8.036403 |
| 199 | 7 | 0.218965 | 0.176448 | 1.532752 | 6.882090 |
| 121 | 7 | 0.211521 | 0.164458 | 1.480646 | 6.648133 |
| 144 | 7 | 0.209211 | 0.163139 | 1.464475 | 6.575525 |
| 36 | 7 | 0.205894 | 0.163928 | 1.441260 | 6.471289 |
| 165 | 7 | 0.191382 | 0.175474 | 1.339672 | 6.015157 |
| 209 | 6 | 0.265719 | 0.248285 | 1.594316 | 7.158514 |
| 219 | 6 | 0.247268 | 0.242944 | 1.483611 | 6.661446 |
| 34 | 6 | 0.183887 | 0.164274 | 1.103320 | 4.953931 |
| 39 | 6 | 0.183426 | 0.159498 | 1.100553 | 4.941507 |
| 105 | 6 | 0.183182 | 0.162876 | 1.099092 | 4.934947 |
| 15 | 6 | 0.162645 | 0.158324 | 0.975869 | 4.381673 |
| 143 | 5 | 0.434621 | 0.324114 | 2.173105 | 9.757289 |
| 104 | 5 | 0.349401 | 0.324114 | 1.747004 | 7.844086 |

In [ ]:

```

```
